# Cdk9 and H2Bub1 signal to Clr6-CII/Rpd3S to suppress aberrant antisense transcription

**DOI:** 10.1093/nar/gkaa474

**Published:** 2020-06-04

**Authors:** Miriam Sansó, Pabitra K Parua, Daniel Pinto, J Peter Svensson, Viviane Pagé, Danny A Bitton, Sarah MacKinnon, Patricia Garcia, Elena Hidalgo, Jürg Bähler, Jason C Tanny, Robert P Fisher

**Affiliations:** Department of Oncological Sciences, Icahn School of Medicine at Mount Sinai, New York, NY, USA; Cancer Genomics Group, Vall d’Hebron Institute of Oncology, Barcelona, Spain; Department of Oncological Sciences, Icahn School of Medicine at Mount Sinai, New York, NY, USA; Department of Pharmacology and Therapeutics, McGill University, Montreal, Canada; Department of Biosciences and Nutrition, Karolinska Institutet, Huddinge, Sweden; Department of Pharmacology and Therapeutics, McGill University, Montreal, Canada; Research Department of Genetics, Evolution & Environment, University College, London, UK; Department of Pharmacology and Therapeutics, McGill University, Montreal, Canada; Departament de Ciènces Experimentals i de la Salut, Universitat Pompeu Fabra, Barcelona, Spain; Departament de Ciènces Experimentals i de la Salut, Universitat Pompeu Fabra, Barcelona, Spain; Research Department of Genetics, Evolution & Environment, University College, London, UK; Department of Pharmacology and Therapeutics, McGill University, Montreal, Canada; Department of Oncological Sciences, Icahn School of Medicine at Mount Sinai, New York, NY, USA

## Abstract

Mono-ubiquitylation of histone H2B (H2Bub1) and phosphorylation of elongation factor Spt5 by cyclin-dependent kinase 9 (Cdk9) occur during transcription by RNA polymerase II (RNAPII), and are mutually dependent in fission yeast. It remained unclear whether Cdk9 and H2Bub1 cooperate to regulate the expression of individual genes. Here, we show that Cdk9 inhibition or H2Bub1 loss induces intragenic antisense transcription of ∼10% of fission yeast genes, with each perturbation affecting largely distinct subsets; ablation of both pathways de-represses antisense transcription of over half the genome. H2Bub1 and phospho-Spt5 have similar genome-wide distributions; both modifications are enriched, and directly proportional to each other, in coding regions, and decrease abruptly around the cleavage and polyadenylation signal (CPS). Cdk9-dependence of antisense suppression at specific genes correlates with high H2Bub1 occupancy, and with promoter-proximal RNAPII pausing. Genetic interactions link Cdk9, H2Bub1 and the histone deacetylase Clr6-CII, while combined Cdk9 inhibition and H2Bub1 loss impair Clr6-CII recruitment to chromatin and lead to decreased occupancy and increased acetylation of histones within gene coding regions. These results uncover novel interactions between co-transcriptional histone modification pathways, which link regulation of RNAPII transcription elongation to suppression of aberrant initiation.

## INTRODUCTION

The elongation phase of transcription by RNA polymerase II (RNAPII) is subject to stringent regulation (1). Among the factors that govern transcription elongation in metazoans are key regulators of cell growth, proliferation and differentiation, including oncogene and tumor-suppressor gene products. Moreover, enzymes involved in RNA processing and chromatin modification are recruited directly to the RNAPII elongation complex, ensuring that these events are coupled to RNA synthesis (2). Despite a growing inventory of proteins and protein modifications implicated in elongation control, many of the underlying molecular mechanisms are still incompletely understood.

Mono-ubiquitylation of histone H2B (H2Bub1) occurs in concert with RNAPII elongation, and is catalyzed by the E2 ubiquitin conjugating enzyme Rad6 and E3 ubiquitin ligases related to *Saccharomyces cerevisiae* Bre1 (3). Consequences of ablating the Bre1 homologs RNF20 and RNF40 in mammalian cells suggest important roles for H2Bub1 in cell growth, differentiation and migration. RNF20, moreover, is often deregulated in patient-derived tumor samples (4–6). H2Bub1 positively regulates methylation of histone H3 at Lys4 (H3K4me) and Lys79 (H3K79me)—marks likewise associated with transcribed chromatin (7,8)—but also functions independently of methylation (9,10), for example, to regulate nucleosome stability and positioning within coding regions (11–13). The mechanisms underlying these methylation-independent effects and their consequences for gene expression are not known. Despite the presence of H2Bub1 at most or all transcribed genes, its loss affects steady-state levels of only a small fraction of mRNAs, suggesting redundant or compensatory homeostatic mechanisms (4,9,11,14).

In yeast and metazoans, co-transcriptional H2Bub1 formation depends on cyclin-dependent kinase 9 (Cdk9), an essential CDK with multiple functions and targets (15–17). In metazoans, Cdk9 is the catalytic subunit of positive transcription elongation factor b (P-TEFb), which releases RNAPII from a promoter-proximal pause—a rate-limiting step in expression of many stringently regulated genes (1). In the fission yeast *Schizosaccharomyces pombe*, Cdk9 activity is needed to overcome an early checkpoint control in transcription and allow RNAPII to attain maximal rates of elongation (18). Near the end of the transcription cycle, protein phosphatase 1 (PP1)-dependent reversal of Cdk9 signaling is needed for timely termination (19). Cdk9 works in part by phosphorylating the elongation factor Spt5, which creates a binding site for Rtf1, an important cofactor for H2Bub1 deposition (20–22). Rtf1 and the H2Bub1-specific E3 ligase Brl2 (ortholog of Bre1) in turn promote Cdk9 recruitment and Spt5 phosphorylation in a positive feedback loop (22,23); an analogous mechanism involving Cdk9 and RNF20 operates in mammalian cells (24). In *S. pombe*, combining an *spt5* mutation that ablates the major phosphorylation sites with deletion of *set1^+^*, which encodes the methyltransferase responsible for H3K4me, recapitulated phenotypes associated with H2Bub1 loss, suggesting that phospho-Spt5 (pSpt5) and Set1 act in parallel pathways downstream of H2Bub1 (23).

Factors that control elongation by RNAPII influence the structure of transcribed chromatin and play key roles in suppressing the aberrant initiation of transcription (sense or antisense) from within gene coding regions. These mechanisms involve cooperation between elongation factors and proteins that modify chromatin structure, including histone chaperones, ATP-dependent chromatin remodelers, and histone-modifying enzymes (25–27). For example, the histone methyltransferase Set2 trimethylates H3 lysine 36 (H3K36me3) in gene coding regions, thereby promoting the function of a histone deacetylase (HDAC) complex (Rpd3S in *S. cerevisiae*, Clr6-CII in *S. pombe*, Sin3B in human cells) and suppressing aberrant transcription initiation (28–30). Although the Cdk9 ortholog Bur1 is linked to activity of both Set2 and Rpd3S in budding yeast (31,32), crosstalk between the Cdk9/H2Bub1 and Set2/HDAC histone modification pathways in the regulation of aberrant transcriptional events has not been studied.

To uncover gene-regulatory mechanisms dependent on Cdk9 and H2Bub1, we analyzed (i) steady-state RNA levels upon loss of H2Bub1, inhibition of Cdk9, or both perturbations, by strand-specific RNA sequencing (RNA-seq); and (ii) distributions of H2Bub1 and pSpt5 on chromatin in unperturbed cells, by chromatin immunoprecipitation and sequencing (ChIP-seq). The most prevalent changes in RNA abundance due to loss of either Cdk9 activity or H2Bub1 were increases in antisense transcripts derived from gene coding regions. The genes at which Cdk9 inhibition alone induced antisense transcription were enriched for ones that have higher-than-average H2Bub1 occupancy and exhibit promoter–proximal RNAPII pausing (33). Genes that depend strictly on H2Bub1 for antisense suppression, conversely, tend to have low H2Bub1 occupancy. When both pathways were impaired, antisense transcription increased at the majority of genes. This combined effect was associated with a genome-wide mismatch between RNAPII and Clr6-CII localization, as well as decreased nucleosome occupancy and increased histone acetylation. Moreover, genetic epistasis tests placed Clr6-CII downstream of both Cdk9 and H2Bub1 with respect to antisense suppression. Therefore, we surmise that Cdk9 and H2Bub1 collaborate to suppress antisense transcription by promoting chromatin-maintenance functions of the Clr6–CII HDAC complex.

## MATERIALS AND METHODS

### Yeast strains and growth conditions

Yeast strains used in this study are listed in Supplementary Table S1. Strain JTB414 (*cph1Δ::kanMX4*) was derived from an *h+/h+* diploid (purchased from Bioneer) after transformation with plasmid pON177 to induce sporulation (34). A hygromycin-resistant version of this strain (JTB622) was derived by PCR-based marker switch as described (35). The *htb1-K119R::hphMX6* allele was similarly derived from strain JTB86-3 (9). Correct marker integration was confirmed by PCR. Double and triple mutants were generated by mating and tetrad dissection. Strains were cultured in YES (5% yeast extract, 30% dextrose, 250 mg/l each of adenine, leucine, histidine, and uracil) at 30°C.

### RNA-seq analysis

*S. pombe* cell cultures (50 mL) of strains JTB362 (WT), JTB425 (*cdk9^as^*), JTB97 (*htb1-K119R*), and JTB508 (*cdk9^as^ htb1-K119R*) were grown to OD_600_ of 0.2 before treatment with DMSO or 20 μM 3-MB-PP1 for 1 h. Total RNA was extracted by a hot phenol method (36) and further purified on Qiagen RNeasy columns. Strand-specific RNA-seq libraries were prepared from poly(A)-enriched RNA as described (37). Fastq files were aligned to the *S. pombe* ASM294v2 genome assembly. Resulting Sam-files were uploaded to Podbat (38) for further analysis. For feature annotation, ASM294v2.20 was used. Data were normalized reads per kilobase per million (RPKM) and averaged over duplicates. Normalization using DESeq (39) yielded broadly similar results (Supplementary Figure S1). Sense transcripts were defined as those that map to annotated protein-coding genes (5123 loci) and antisense transcripts as those that map antisense to annotated genes (those that overlapped more than one gene were discarded). Intergenic transcripts were defined as those that map between annotated coding and non-coding loci. RPKM for each feature was calculated separately for sense and antisense. To determine differentially expressed genes, we used a cut-off of 2-fold (values in log 2 space >1 or < -1), with the highest absolute value being above 1 RPKM to filter out noise.

### Quantitative RT-PCR (RT-qPCR)

For strand-specific RT-qPCR to measure levels of *cdc2^+^, erg32^+^, hrp1^+^* and *hem2^+^* antisense transcripts, 1–5 μg total RNA was converted to cDNA using the RevertAid H minus kit (Invitrogen) and gene-specific primers listed in Supplementary Table S2. Expression levels were normalized to those of the *act1*^+^ sense transcript in each strain.

### Chromatin immunoprecipitation (ChIP)

ChIP was carried out as described previously (23,40). Briefly, *S. pombe* cell cultures were grown in YES to OD_600_ of 0.3–0.6, treated with DMSO or 20 μM 3-MB-PP1 for 20 min, and crosslinked with 1% formaldehyde for 15 min at room temperature. To terminate crosslinking, 2.5 M glycine was added to a final concentration of 125 mM for 5 min. Cells were pelleted by centrifugation, washed twice with 10 mL cold TBS (10 mM Tris pH 7.5, 150 mM NaCl), frozen on dry ice and stored at –80°C. Cell pellets from 50 ml cultures were resuspended in 0.4 ml FA lysis buffer [50 mM HEPES pH 7.6, 150 mM NaCl, 1 mM EDTA, 1% Triton X-100, 0.1% sodium deoxycholate, 1 mM PMSF and protease inhibitor cocktail (Roche)] and lysed in a mini-bead beater (Biospec products) in the presence of glass beads (50 micron; Sigma) at 4°C for 2 min. Lysates were centrifuged at 16,100 × g_av_ for 15 min at 4°C. Pellets containing chromatin were resuspended in 1 ml FA Lysis buffer and transferred to 15 ml polycarbonate tubes. Lysates were sonicated for 20 min at 4°C (30 s on, 30 s off, output setting high) using a waterbath sonicator (Diagenode Bioruptor), transferred to new 1.5-ml tubes and centrifuged at 16,100 × g_av_ for 5 min at 4°C. 100 μl of supernatant was kept as ‘input’ and the remainder (∼900 μl) was subjected to immunoprecipitation with antibodies against histone H3 (Abcam 1791), K9,14-acetylated histone H3 (Millipore 06-599), K36 trimethylated histone H3 (Abcam 9050), FLAG epitope (M2, Sigma), or control IgG. Lysates prepared in parallel from *S. cerevisiae* strains BY4743 or a derivative expressing Rpb1-FLAG were spiked in to each IP (10% of lysate volume) for normalization. After incubation at 4°C with mixing for ≥4 h, immune complexes were recovered using 15 μl protein A/G agarose beads (Thermo Fisher) and incubated for an additional hour. Beads were washed sequentially with 0.5 ml FA Lysis + 0.1% SDS, FA Lysis + 0.1% SDS + 500 mM NaCl, LiCl buffer (10 mM Tris pH 7.5, 1 mM EDTA, 250 mM LiCl, 0.5% sodium deoxycholate, 0.5% NP-40), and TE (10 mM Tris pH 7.5, 1 mM EDTA). Each wash was for 4 min with mixing at room temperature. Immune complexes were eluted in 100 μl elution buffer (50 mM Tris pH 7.5, 10 mM EDTA, 1% SDS) at 65°C for 20 min. Beads were washed with 150 μl TE + 0.67% SDS, which was combined with the eluate. 150 μl TE + 0.67% SDS was also added to the input samples, and both IP and input samples were incubated at 65°C overnight to reverse protein–DNA crosslinks. DNA was purified by phenol/chloroform extraction as described previously (23). Analysis by qPCR was carried out using a Bio-Rad CFX96 instrument, Bio-Rad iQ Green SYBR mix, and primers listed in Supplementary Table S2. ChIP signals were calculated as IP/input and normalized to *S. cerevisiae* spike-in.

ChIP-seq analysis was carried out in a strain expressing FLAG-tagged histone H2B (strain MS265) using an H2Bub1-specific antibody (Active Motif) and anti-FLAG antibody (Sigma). Total Spt5 and phospho-Spt5 were immunoprecipitated from a Myc-tagged Spt5 strain (strain CS111) using anti-Myc antibody (EMD Millipore) and a previously described pSpt5-specific antibody (23), respectively, and analyzed as described previously (21). Briefly, multiplexed ChIP-seq libraries were prepared using the NEBNext® Ultra™ II DNA- and Illumina TruSeq DNA-Library Preparation kit with 25–75 ng of input or IP DNA and barcode adaptors. Paired-end sequencing (50-nt reads) was performed on an Illumina NextSeq 500 and Illumina HiSeq 2000. After adaptor trimming and quality control, processed FASTQ files were aligned to the *S. pombe* genome using Bowtie2 (Galaxy Version 2.2.6.2). Aligned sequences of each biological replicate were fed into MACS2 (Galaxy Version 2.1.1.20160309.0) to call peaks from alignment results. Generated ‘bedgraph treatment’ files were concatenated (Galaxy Version 1.0.1) to combine replicates of each sample, converted into bigwig using ‘Wig/BedGraph-to-bigWig converter’ (Galaxy Version 1.1.0) and processed using computeMatrix (Galaxy Version 2.3.6.0) in DeepTools to prepare data for plotting heatmaps and/ or profiles of given regions. Heatmap and Metagene plots were generated using ‘plotHeatmap’ (Galaxy Version 2.5.0.0) and ‘plotProfile’ (Galaxy Version 2.5.0.0) tools, respectively. The pSpt5: Spt5 and H2Bub1: H2B signal ratios were calculated using bigwigCompare (Galaxy Version 2.5.0.0).

ChIP-seq of Clr6-CII and RNAPII was performed in strains expressing *pst2-myc* using anti-myc antibody (BioLegend # 626802) and 8WG16 (BioLegend # 664912), which recognizes the Rpb1 carboxy-terminal domain (CTD), respectively. Lysates from *S. cerevisiae* strain BY4743 expressing TFIIB-myc were spiked in to IPs (1% total lysate volume) for normalization, which was applied after alignment of FASTQ files to both *S. cerevisiae* and *S. pombe* genomes.

## RESULTS

### Cdk9 and H2Bub1 collaborate to suppress antisense transcription

**Figure 1.**
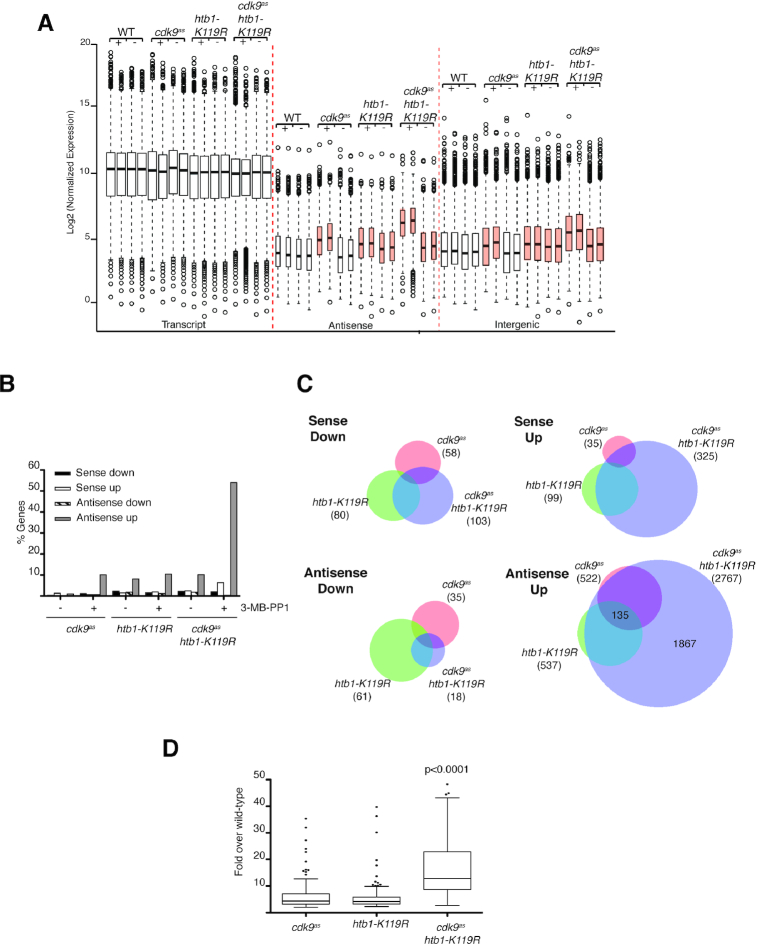
Cdk9 inhibition and *htb1-K119R* synergistically increase antisense transcription. (**A**) Box-and-whisker plots of normalized expression levels for transcripts in the indicated categories for the indicated strains (JTB362, JTB299, JTB86, JTB508). ‘+’ denotes treatment with 3-MB-PP1; ‘−’ denotes treatment with DMSO. Orange boxes highlight increased levels of antisense and intergenic transcripts relative to wild-type. (**B**) Plot of the percentage of protein-coding genes in the indicated categories, defined by a minimum 2-fold change from wild-type expression level in each condition (see Materials and Methods). (**C**) Venn diagrams comparing differentially expressed transcripts (>2-fold change from wild-type) for the indicated strains in the presence of 3-MB-PP1. Numbers in brackets indicate total number of differentially expressed transcripts in indicated strains. (**D**) Box-and-whisker plot of fold-induction (in presence of 3-MB-PP1) for 135 antisense transcripts regulated by Cdk9 activity and H2Bub1. Significance of increased antisense expression levels in the double mutant was determined using the Mann-Whitney test.

To uncover changes in RNAPII transcription that might illuminate previously described genetic interactions between the Cdk9 and H2Bub1 pathways, we performed strand-specific RNA-seq analysis, comparing wild-type cells and ones with 1) an analog-sensitive (*as*) mutation (*cdk9^as^*) that confers sensitivity to inhibition by the bulky adenine analog 3-MB-PP1 (36), 2) *htb1-K119R* or 3) both mutations (41). Cdk9 inhibition or H2Bub1 ablation had specific rather than global effects on steady-state mRNA abundance (Figure 1A), consistent with previous analyses (9,36). We also detected 3-MB-PP1-dependent increases in antisense and intergenic transcript abundance in *cdk9^as^* cells, and drug-independent increases of similar magnitude in *htb1-K119R* cells. Finally, the *cdk9^as^ htb1-K119R* mutant resembled the *htb1-K119R* single mutant in the absence of drugs but, upon addition of 3-MB-PP1, had the highest levels of antisense and intergenic transcripts of any of the four strains.

Individually, either inhibition of Cdk9 or loss of H2Bub1 affected steady-state mRNA levels of <5% of *S. pombe* genes but increased antisense transcript levels of ∼10% of genes (Figure 1B and Supplementary Figure S1 and Table S3). There was significant overlap between the antisense transcripts increased in each of the single-mutant strains, for which the common set comprised ∼25% of genes affected by either mutation alone (*P* < 10^−15^; hypergeometric test), but not for the other classes of affected transcripts (Figure 1C). Cdk9 inactivation in an *htb1-K119R* background led to increased sense transcript accumulation for <10% of genes, whereas antisense transcripts were increased for >50% of genes (Figure 1B and Supplementary Figure S1). The latter group comprised the 135 genes similarly affected by either *htb1-K119R* or Cdk9 inhibition, most of those affected in one case but not the other, and nearly 2000 additional genes (Figure 1C). Quantification of individual transcripts revealed synergistic effects of Cdk9 inhibition and H2Bub1 loss; for genes sensitive to either perturbation, median induction relative to wild type was ∼4-fold in *htb1-K119R* or 3-MB-PP1-treated *cdk9^as^* cells, but ∼12-fold in the drug-exposed double mutant (Figure 1D). Therefore, whereas distinct subsets of genes have non-redundant requirements for Cdk9 or H2Bub1, the majority of protein-coding genes depend on one of the two pathways being intact to suppress antisense transcription.

**Figure 2.**
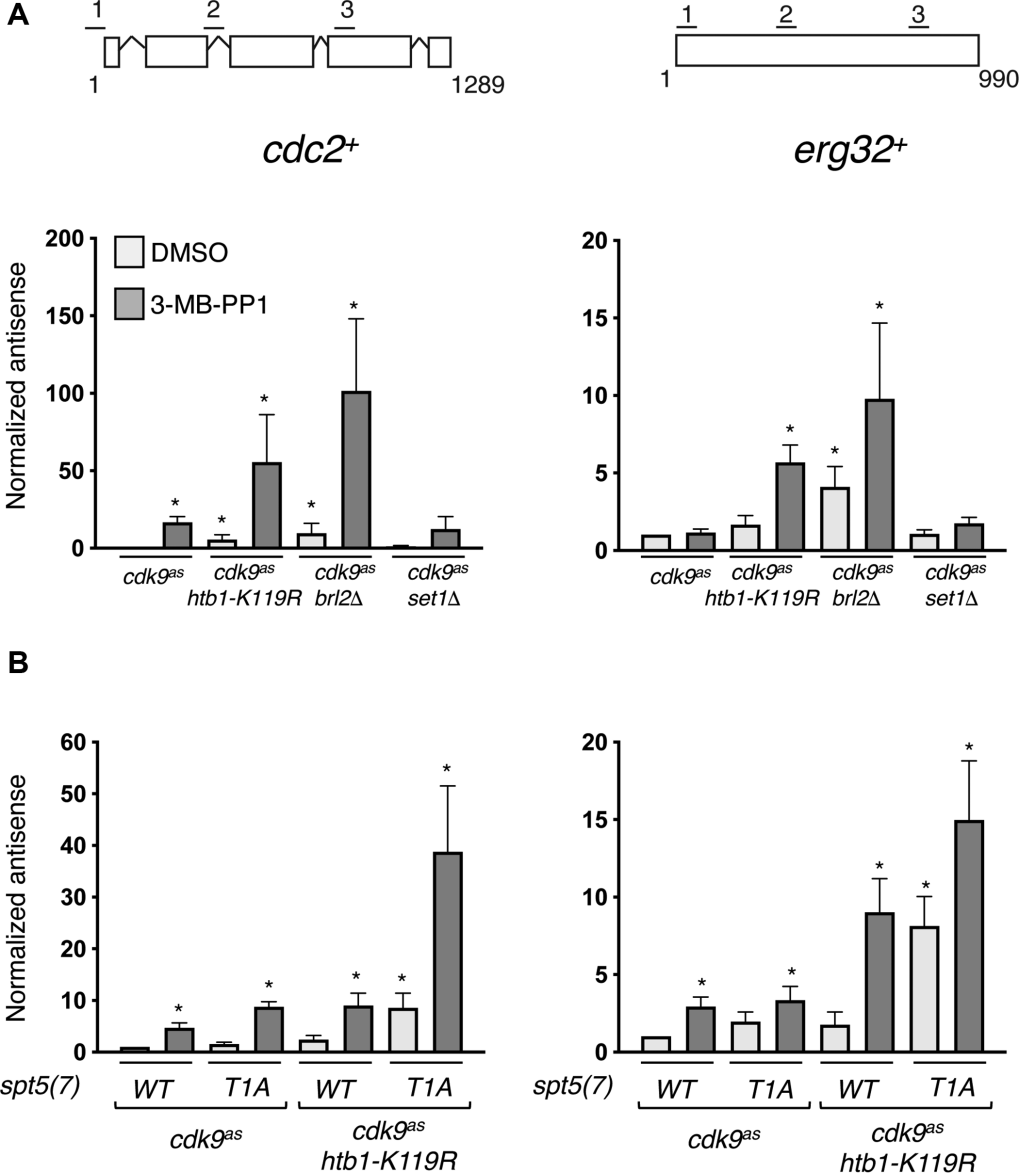
Antisense induction by Cdk9 inhibition and *htb1-K119R* is independent of H3K4me and enhanced by *spt5-T1A*. (**A**) Antisense transcript levels at *cdc2^+^* and *erg32^+^* loci were quantified by strand-specific RT-qPCR in indicated strains (JTB425, JTB508, JTB386, JTB408, respectively). Error bars denote S.D.; ‘*’ denotes significant differences (*P*< 0.05; paired *t*-test) from the DMSO-treated *cdk9^as^* strain (whose value was set to 1; *n* = 3). (**B**) As in (A) for the indicated set of strains (JTB443, JTB444, JTB738-2, JTB736, respectively).

We validated the RNA-seq results by strand-specific RT-qPCR at two loci, *cdc2^+^* and *erg32^+^*; there were relative increases in antisense transcription from *cdc2^+^* due to either Cdk9 inhibition or *htb1-K119R*, and a larger increase when both Cdk9 activity and H2Bub1 were blocked. The effect was more clearly synergistic at *erg32*^+^, where either perturbation alone had only minimal effects (Figure 2A). The *cdc2^+^* antisense transcript corresponded to a previously annotated non-coding RNA (ncRNA), as confirmed by specific RT-PCR (Supplementary Figure S2A) and by blot hybridization with a strand-specific probe (Supplementary Figure S2B).

### Antisense suppression is H3K4me-independent and involves multiple Cdk9 targets

To determine if antisense derepression was due to loss of H2Bub1 per se, we measured transcript levels in a strain that lacked the E3 ligase Brl2. A *brl2Δ* mutation led to increased antisense transcript levels, which were augmented by Cdk9 inhibition, at both *cdc2^+^* and *erg32*^+^, indicating that antisense induction due to *htb1-K119R* is likely to stem from loss of ubiquitylation, rather than another effect of the Lys→Arg substitution (Figure 2A). In contrast, a *set1Δ* mutation that removes the H3K4 methyltransferase Set1 did not influence antisense transcription of either gene, arguing that antisense regulation is an H3K4me-independent function of H2Bub1 (Figure 2A).

We next sought to implicate Spt5, a known target of Cdk9, in antisense suppression. The majority of Spt5 phosphorylation by Cdk9 occurs within a carboxy-terminal domain (CTD) comprising 18 repeats of a nonapeptide motif (consensus: T_1_P_2_A_3_W_4_N_5_S_6_G_7_S_8_K_9_) (36). Within the repeats, Cdk9 phosphorylates the Thr1 position in vitro (42), and this phosphorylation is lost within seconds after inhibition of Cdk9 in vivo (18,23). We analyzed antisense transcript levels in strains expressing Spt5 variants with only seven repeats, a truncation that did not by itself compromise cell growth or viability (43). A Thr→Ala substitution of the first position in each repeat—*spt5(T1A*)*_7_*–did not increase antisense transcription of *cdc2^+^* or *erg32*^+^ in an *htb1^+^* strain, but it produced levels of antisense transcripts similar to those caused by Cdk9 inhibition in an *htb1-K119R* background (Figure 2B), suggesting that the Spt5 CTD is a critical target of Cdk9 in antisense suppression. However, *spt5(T1A*)*_7_* strains remained sensitive to Cdk9 inhibition; treatment of *cdk9^as^ htb1-K119R spt5(T1A*)*_7_* cells with 3-MB-PP1 produced the highest levels of antisense transcripts detected at either gene. Therefore, whereas constitutive loss of pSpt5 is not sufficient to derepress *cdc2^+^* or *erg32^+^*antisense transcription in H2Bub1-proficient cells, it sensitizes cells to antisense induction by H2Bub1 loss, *and* to combined H2Bub1 loss and Cdk9 inhibition. This suggests that Cdk9 inhibition might derepress antisense transcription by more than one mechanism, possibly by affecting multiple Cdk9 substrates.

### High H2Bub1 and pausing predict Cdk9-dependence of antisense suppression

**Figure 3.**
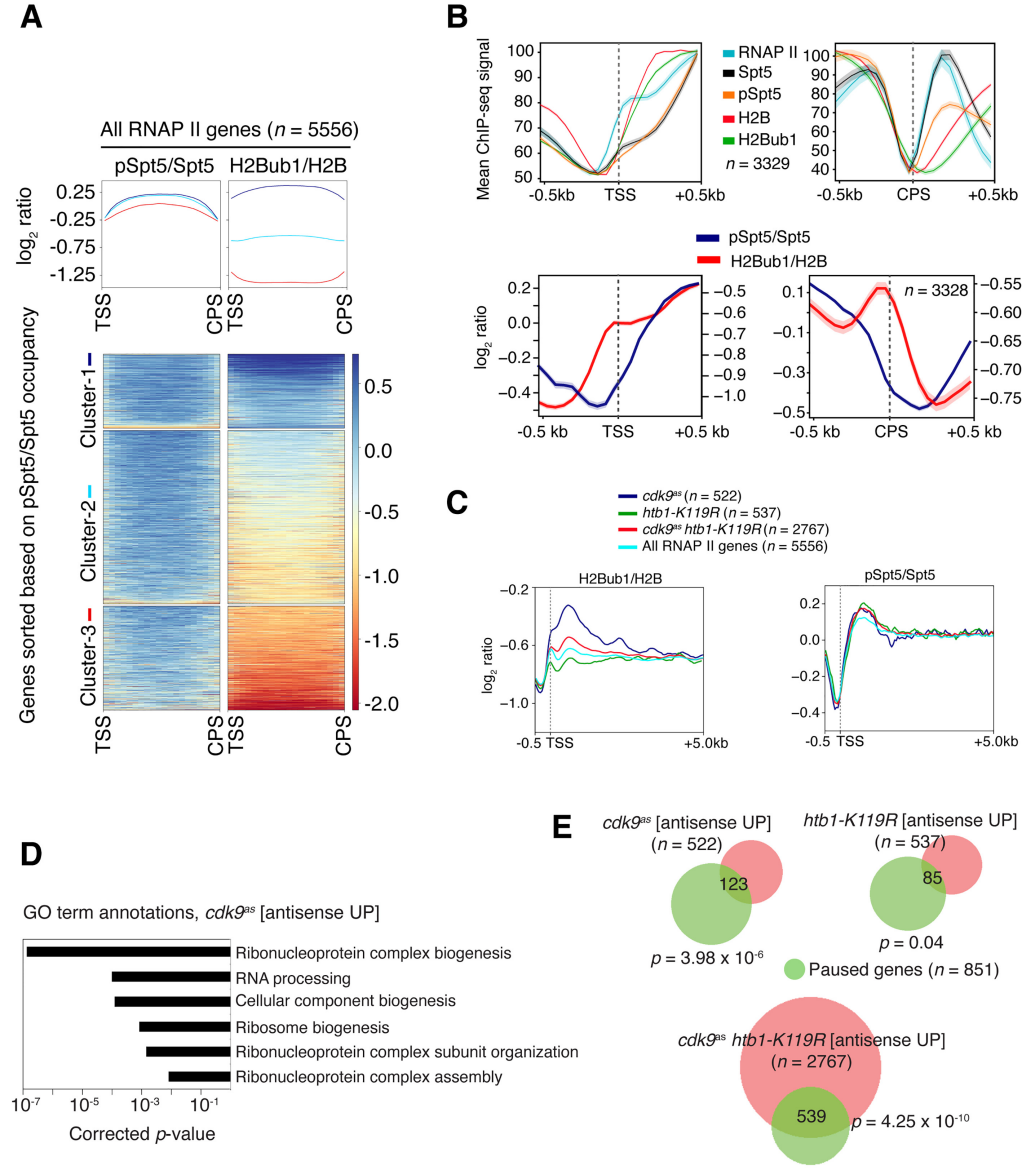
H2Bub1 levels are proportional to pSpt5 and correlated with antisense regulation by Cdk9 and RNAPII promoter-proximal pausing. (**A**) Top: Scaled metagene representations of pSpt5/Spt5 or H2Bub1/H2B ratios from transcription start site (TSS) to cleavage and polyadenylation site (CPS) for all genes. Genes were clustered based on total pSpt5/Spt5 occupancy. Bottom: Heat map representation of pSpt5/Spt5 and H2Bub1/H2B ratios for genes in each cluster. (**B**) Top: Metagene plots show the distribution of RNAPII, Spt5, pSpt5, H2B, and H2Bub1 around the TSS and CPS (±500 bp) for *n* = 3329. Bottom: Plots show the distribution of log_2_-ratio of pSpt5/Spt5 and H2Bub1/H2B around the TSS and CPS (±500 bp) for the same set of genes (*n* = 3329). (**C**) Metagene representations of H2Bub1/H2B or pSpt5/Spt5 ratios for the indicated groups of genes, plotted from the indicated distances (in kilobases) relative to the TSS. (**D**) Gene ontology (GO) terms enriched among genes in the ‘*cdk9^as^* antisense UP group’ (Fisher's exact test with Bonferroni correction). (**E**) Venn diagrams indicating overlap between the indicated gene groups and genes with promoter-proximal RNAPII pausing (hypergeometric test).

To understand how Cdk9 and H2Bub1 cooperate to suppress antisense transcription, we compared genome-wide distributions of pSpt5 (19) and H2Bub1, determined by ChIP-seq analysis in wild-type cells. As expected, given their mutual dependence in vivo (23), occupancy of both H2Bub1 and pSpt5 was maximal in gene coding regions. Levels of H2Bub1 at individual genes, moreover, were directly proportional to those of pSpt5; hierarchical clustering of *S. pombe* genes based on pSpt5:Spt5 ratio sorted them by H2Bub1:H2B ratio as well (Figure 3A). Interestingly, pSpt5:Spt5 varied relatively little among genes, whereas H2Bub1:H2B varied over a wider range (Figure 3A and Supplementary Figure S3A–C). This greater dynamic range suggests that H2Bub1 is the more sensitive indicator of active transcription, notwithstanding its mechanistic coupling to pSpt5 (22,23). Near the transcription start site (TSS), H2Bub1 increased both in absolute terms and relative to total H2B, roughly in tandem with RNAPII and in advance of the increase in pSpt5 (Figure 3B). Near the 3′ end, the sharp drop in pSpt5:Spt5 just upstream of the CPS—which is dependent on the PP1 isoform Dis2 (44)—was accompanied by an *increase* in H2Bub1:H2B, which was entirely due to a decrease in total histone H2B occupancy (Figure 3B). Downstream of the CPS, H2B crosslinking increased again and the H2Bub1:H2B ratio dropped, suggesting the replacement of modified by unmodified nucleosomes.

The proportional relationship between pSpt5:Spt5 and H2Bub1:H2B held when we performed similar clustering analysis of genes that gave rise to increased antisense transcription when Cdk9 was inhibited, H2Bub1 was prevented, or both pathways were blocked (Supplementary Figure S3A). Strikingly, however, the genes that depend strictly on Cdk9 for antisense suppression had higher levels of H2Bub1 occupancy than did the other two sets (Figure 3C, Supplementary Figures S3A and S3B). Conversely, the set that required H2Bub1 to prevent antisense transcription had lower levels of this histone mark than did the other sets. No similar differences among gene sets were evident in metagene or heatmap analysis of pSpt5:Spt5 (Figure 3C and Supplementary Figure S3C). By gene ontology (GO) term enrichment analysis, the Cdk9-dependent set (‘*cdk9^as^* [antisense UP]’) was enriched for functions involved in ribonucleoprotein complex biogenesis, RNA processing and ribosome biogenesis (Figure 3D). The same GO terms were enriched among genes with promoter–proximally paused RNAPII, previously defined by precision run-on transcription and sequencing (PRO-seq) analysis (33). There was statistically significant overlap of the paused genes with ‘*cdk9^as^* [antisense UP]’ and ‘*cdk9^as^ htb1-K119R* [antisense UP]’ sets defined by RNA-seq (Figure 3E). Taken together, the data suggest a functional relationship between pausing and a Cdk9 requirement to suppress unscheduled initiation within gene bodies.

### H2Bub1 and Cdk9 interact genetically with the Clr6-CII complex

**Figure 4.**
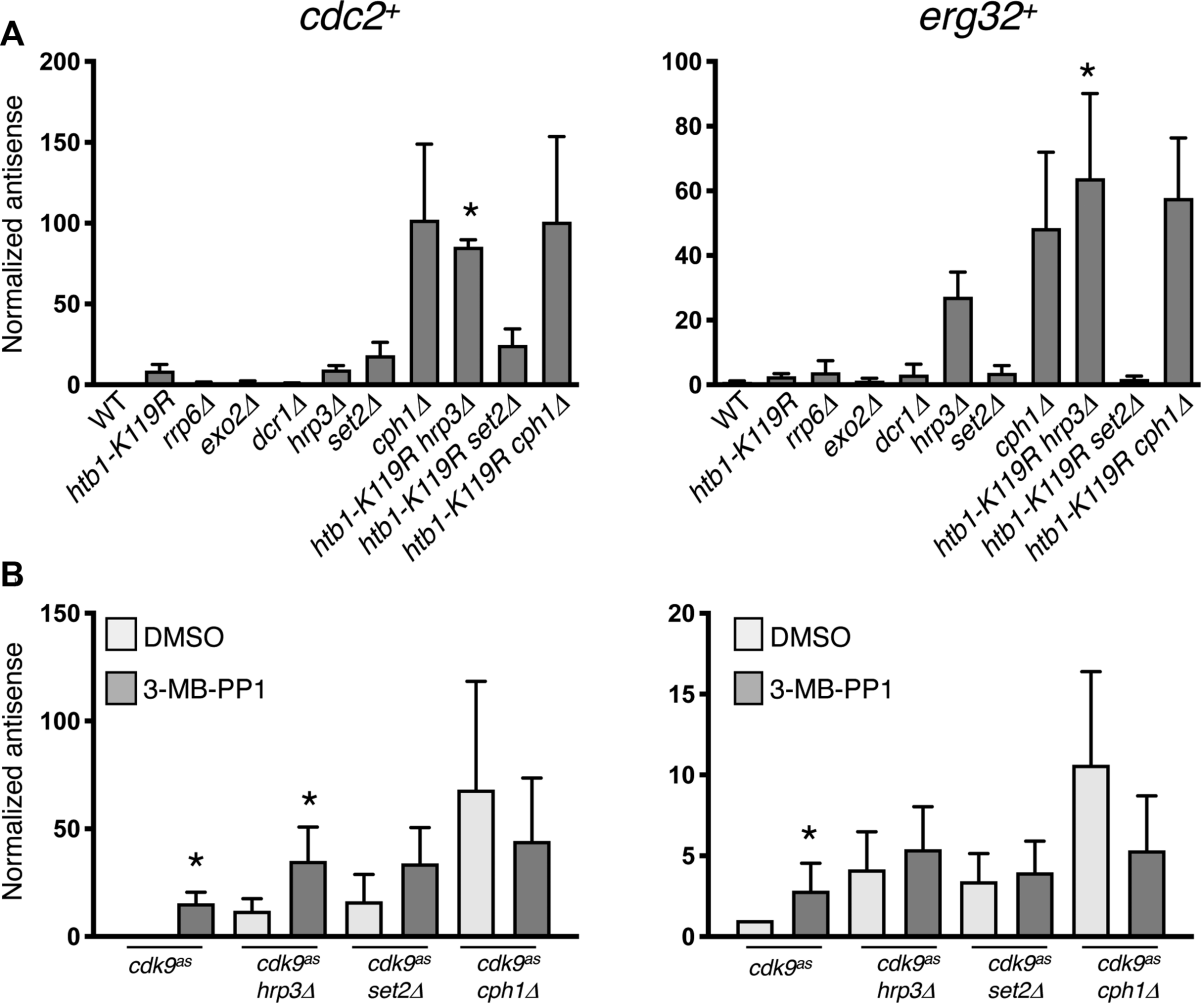
Epistasis relationships implicate Set2 and the Clr6-CII HDAC complex downstream of Cdk9 and H2Bub1. (**A**) Antisense transcript levels at *cdc2^+^* and *erg32^+^* loci were quantified by strand-specific RT-qPCR in the indicated strains. Error bars denote S.D. from 3–4 independent experiments; ‘*’ denotes significant differences (*P*< 0.05; paired *t-test*) between the indicated double mutant strain and the respective single mutants. Value for the wild-type strain was set to 1. (**B**) As in (A) for the indicated *cdk9^as^* strains. ‘*’ denotes significant differences (*P*< 0.05; unpaired *t*-test) between 3-MB-PP1-treated cells and the corresponding DMSO-treated control. Value for the DMSO-treated *cdk9^as^* strain was set to 1.

Previous studies in fission yeast identified two classes of factors involved in regulation of antisense transcription: (i) those that work by modifying chromatin, such as Clr6-CII (29,45) and (ii) those that target antisense transcripts for post-transcriptional elimination such as the nuclear exosome, the cytoplasmic exonuclease Exo2 (ortholog of XRN1), and the dsRNA-specific nuclease Dicer, encoded by *dcr1^+^* (27,28,46,47). To ask if either of these mechanisms is involved in antisense suppression by Cdk9 and H2Bub1, we analyzed mutant strains lacking representative factors from each class. Consistent with a chromatin-based mechanism, *cdc2^+^*and *erg32*^+^ antisense transcript levels were increased by deletion of *cph1^+^* (encoding a subunit of Clr6-CII (27,28)), *set2*^+^, or *hrp3^+^* (encoding a chromatin-remodeling factor (48–50)) (Figure 4A). Deletion of *rrp6*^+^ (a component of the nuclear exosome), *exo2*^+^ or *dcr1*^+^ did not increase either antisense transcript, indicating that they are not regulated post-transcriptionally. These results point to regulators of chromatin organization—Clr6-CII, Set2 and Hrp3—as candidate effectors of antisense suppression by Cdk9 and H2Bub1.

Metagene analysis of antisense transcripts induced in 3-MB-PP1-treated *cdk9^as^ htb1-K119R* cells revealed uniform distribution of signals throughout gene coding regions (Supplementary Figure S4A). Therefore, most of these antisense transcripts arose from within the bodies of transcribed genes, as was the case in mutants affecting Clr6-CII and related factors (25,49,51). There was no apparent bias towards adjacent, convergently transcribed gene pairs, which might give rise to read-through antisense transcripts due to inefficient termination, further supporting an intragenic origin (Supplementary Figure S4B).

To understand interactions between H2Bub1 and components of other chromatin modification pathways (Clr6-CII, Set2 and Hrp3), we measured *cdc2*^+^ and *erg32*^+^ antisense levels in double-mutant strains. At *cdc2*^+^, *htb1-K119R* and *hrp3Δ* each increased antisense levels by ∼5- to 10-fold, whereas the combination enhanced antisense transcription by ∼100-fold over wild-type levels (Figure 4A). The strong synergy argues that H2Bub1 and Hrp3 function in separate pathways. The antisense effects were independent of effects on sense transcript levels, which varied by ∼2–3-fold in the same strains (Supplementary Figure S5A). A similar synergy was apparent at *erg32^+^* and at *hrp1^+^*, a gene known to express antisense transcripts in mutants defective in chromatin structure (28,49) (Figure 4A and Supplementary Figure S5B). In contrast, antisense-inducing effects of *htb1-K119R* and *cph1Δ*, or of *htb1-K119R* and *set2Δ*, were not additive at these genes or at *hem2^+^* (a previously characterized H2Bub1 target gene), indicating genetic epistasis (Figure 4A, Supplementary Figures S5B and S5C) (9). Similarly, in a *cph1Δ* background Cdk9 inhibition did not change (or slightly decreased) antisense levels, whereas inhibition of Cdk9 in combination with either *hrp3Δ* or *set2Δ* led to effects on antisense transcription that varied, i.e., were either additive or epistatic, at different genes (Figure 4B, Supplementary Figures S5D and S5E). Taken together, the epistasis relationships and intragenic origins of antisense transcripts suggest that Set2 and H2Bub1 work through the same pathway, and that Clr6-CII is a common effector of antisense suppression by Cdk9 and H2Bub1.

### Cdk9 and H2Bub1 promote chromatin association of Clr6-CII

**Figure 5.**
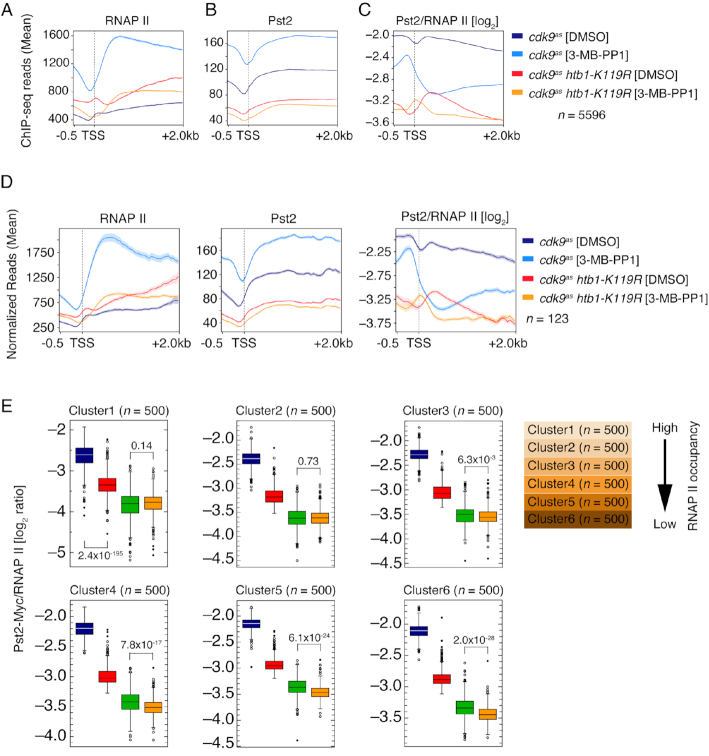
Chromatin occupancy of Clr6-CII is regulated by Cdk9 activity and H2B ubiquitylation. (**A**, **B** and **C**) Distribution of RNAPII, Pst2, and their log_2_-ratio (Pst2/RNAPII) upstream (–0.5 kb) and downstream (+2 kb) of the TSS, respectively, for all RNAPII-activated genes (*n* = 5596) in the indicated strains after treating with 3-MB-PP1 or DMSO. (**D**) Left to right: Metagene distribution of RNAPII, Pst2, and their log_2_-ratio (Pst2/RNAPII) upstream (−0.5 kb) and downstream (+2 kb) of the TSS for paused genes that overlapped with the genes that showed increased antisense transcription upon acute inhibition of Cdk9 (Figure 3E; *n* = 123). (**E**) Box plots showing effects of perturbation of Cdk9 function and H2B ubiquitylation individually and in combination on Pst2/RNAPII log2-ratio calculated on individual genes. Clusters are 500 genes each and are ordered based on RNAPII occupancy, high to low. Blue-*cdk9^as^*[DMSO]; red-*cdk9^as^*[3-MB-PP1]; green-*cdk9^as^htb1*-*K119R* [DMSO]; orange-*cdk9^as^htb1*-*K119R* [3-MB-PP1].

Uniquely among the genes we tested, *cph1^+^*—encoding a subunit of the Clr6-CII HDAC complex—was epistatic with both *htb1-K119R* and *cdk9^as^* at all four loci. Therefore, to ask if H2Bub1 loss or Cdk9 inactivation affected Clr6-CII recruitment to transcribed chromatin, we performed parallel ChIP-seq analyses of RNAPII and the Clr6-CII subunit Pst2 in *pst2-myc* strains. Metagene analysis revealed that Cdk9 inhibition caused a shift of RNAPII density toward the 5′ ends of gene coding regions and enhanced RNAPII occupancy throughout gene bodies by ∼3-fold (Figure 5A). These effects are likely due to impaired Cdk9 function in allowing RNAPII to escape an early elongation checkpoint and attain a maximal rate of elongation (18). Absolute levels of Pst2 occupancy also increased in gene bodies, but the Pst2:RNAPII ratio was decreased (Figure 5B and C), suggesting that active Cdk9 is needed for coupling of Clr6-CII function with transcription. The relative depletion of Pst2 occurred genome-wide despite unchanged bulk Pst2 levels measured by immunoblotting (Supplementary Figure S6A), and was not limited to genes for which antisense transcripts were increased by Cdk9 inhibition (Supplementary Figure S6B-D), indicating that reduced Pst2 occupancy was not sufficient to cause aberrant antisense transcription. However, among the groups of genes we analyzed, the effect on Pst2 recruitment was most severe on paused genes that responded to Cdk9 inhibition with increased antisense transcription (Figure 5D). Cdk9 inhibition did not dramatically alter the intragenic distribution of Pst2 either genome-wide or on this select subset of genes, despite the 5′ shift of RNAPII distribution (Figure 5A, B and D). Therefore, Cdk9 inhibition leads to both quantitative and spatial uncoupling of Clr6-CII and RNAPII occupancy on transcribed chromatin.

Loss of H2Bub1 led to enhanced RNAPII occupancy in gene bodies, based on a comparison between *cdk9^as^* and *cdk9^as^ htb1-K119R* cells, both treated with DMSO (Figure 5A)—a condition in which *cdk9^as^* and wild-type cells had indistinguishable active RNAPII distributions by PRO-seq analysis (18). In addition, there was a re-distribution of RNAPII towards the 3′ ends of genes in the double mutant, as previously detected by ChIP-chip analysis (23), and a peak of RNAPII occupancy that appeared near the TSS. Pst2 occupancy was diminished ∼2-fold in DMSO-treated *cdk9^as^ htb1-K119R* compared to *cdk9^as^* cells, again with no effect on its intragenic distribution (Figure 5B and C). Either H2Bub1 loss or Cdk9 inhibition alone was associated with highly significant decreases in Pst2:RNAPII ratio affecting all genes, irrespective of absolute levels of RNAPII occupancy (and, we infer, expression levels), with the *htb1-K119R* mutation producing the larger diminution (Figure 5E). As was the case for Cdk9 inhibition, there was no preferential effect on Pst2 recruitment to genes in which antisense transcripts were increased by *htb1-K119R* (Supplementary Figure S6C). Treatment of *cdk9^as^ htb1-K119R* cells with 3-MB-PP1 decreased the 5′ and 3′ accumulation of RNAPII, but did not result in the global increase in RNAPII occupancy observed in the *cdk9^as^* strain (Figure 5A). In addition, Pst2:RNAPII ratio was further decreased relative to DMSO-treated, H2Bub1-deficient cells (Figure 5C). This reduction was smaller than that due to Cdk9 inhibition in *htb1^+^* strains, and was only significant for the 2,000 genes with the lowest RNAPII occupancy (Figure 5E). Thus, both Cdk9 and H2Bub1 were individually required for Clr6-CII association with transcribed chromatin, and their combined loss further decreased Clr6-CII occupancy within gene bodies.

The epistasis relationships suggested that H2Bub1 and Set2 regulate antisense transcription through the same pathway, whereas Cdk9 acted independently of Set2 at certain genes such as *cdc2^+^*. We thus measured the effect of Cdk9 inhibition and *htb1-K119R* on chromatin association of Set2 and levels of H3K36 methylation by ChIP-qPCR analysis (Supplementary Figures S7A and S7B). The *htb1-K119R* mutation on its own had no significant effect on Set2 recruitment or H3K36me at *cdc2^+^* and *erg32^+^*, suggesting it regulates antisense transcription at a step downstream of H3K36me. In contrast, comparison of DMSO- to 3-MB-PP1-treated *cdk9^as^* strains (either *htb1^+^* or *htb1-K119R*) indicated a requirement for Cdk9 activity for Set2 recruitment at both genes. However, there were no effects on H3K36me levels (relative to total H3), likely due to slow turnover of this histone mark (40). We conclude that decreased Set2 activity does not account for the synergistic effect of Cdk9 inhibition and *htb1-K119R* on antisense regulation.

**Figure 6.**
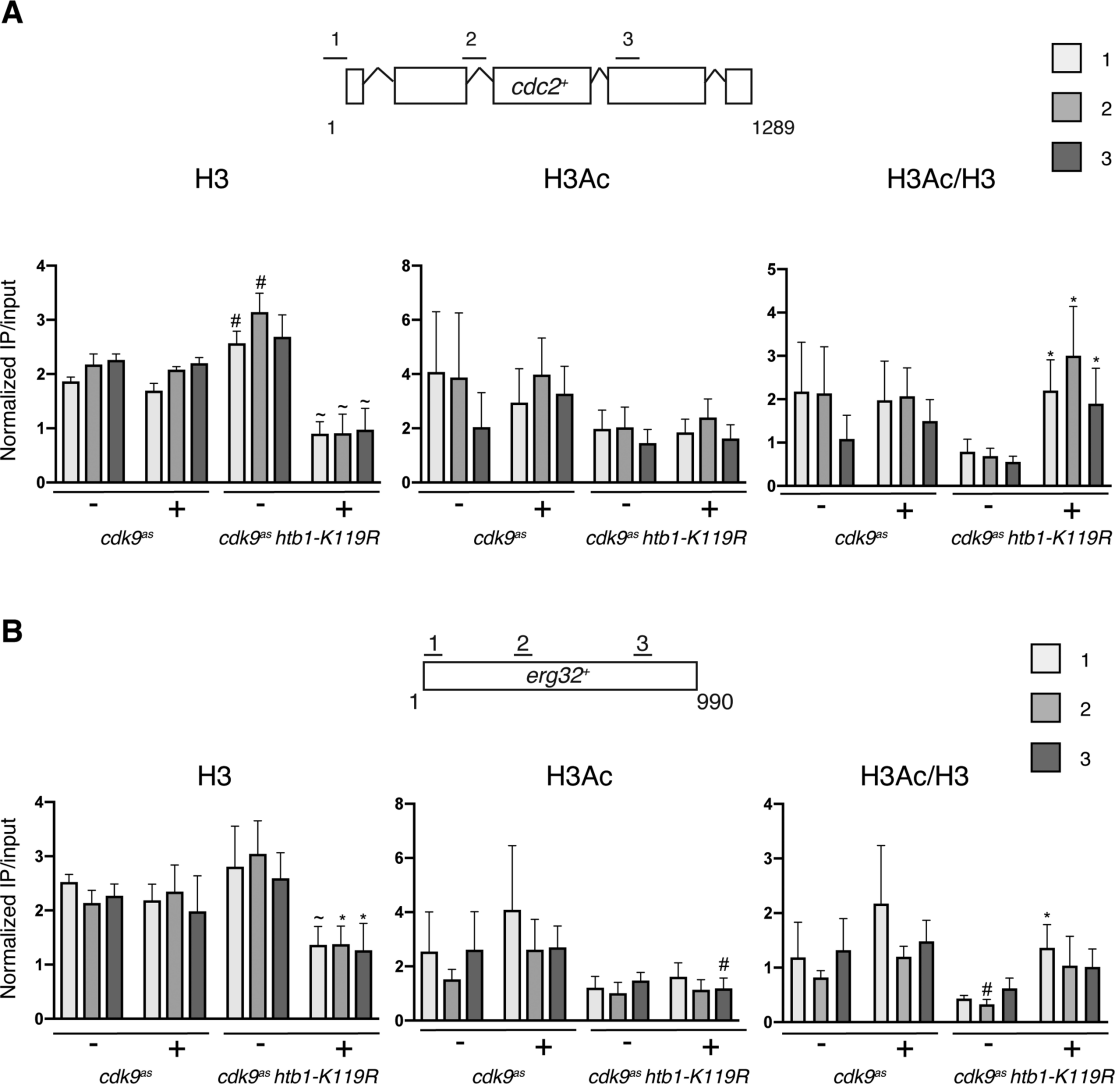
Antisense induction in *cdk9^as^ htb1-K119R* correlates with increased histone acetylation and decreased histone occupancy. (**A**) ChIP-qPCR was performed with the indicated antibodies (top) and quantified with primer pairs at the indicated positions in *cdc2*^+^ (see Materials and Methods). ‘–‘ and ‘+’ denote treatment with DMSO or 3-MB-PP1, respectively. Error bars denote S.D. from 3–4 independent experiments. ‘*’ denotes a significant difference (*P*<0.05; unpaired *t*-test) between treatments (DMSO or 3-MB-PP1) for a given strain; ‘#’ denotes a significant difference between strains for a given treatment; ‘∼’ denotes significant differences in both comparisons. (**B**) As in (A) for *erg32*^+^.

Having established that H2Bub1 and Cdk9 regulate Clr6-CII occupancy, we asked if H2Bub1 loss or Cdk9 inhibition impaired Clr6-CII function, which might be reflected by changes in histone occupancy and/or acetylation. ChIP-qPCR at *cdc2^+^* and *erg32^+^*showed no significant difference between histone H3 occupancy in DMSO- versus 3-MB-PP1-treated *cdk9^as^* cells (Figure 6A and B). Comparison of *cdk9^as^* to *cdk9^as^ htb1-K119R* (both DMSO-treated) revealed a slight increase in H3 occupancy in the latter. In contrast, 3-MB-PP1 treatment of the *cdk9^as^ htb1-K119R* strain led to significantly decreased H3 crosslinking (2–3-fold) at both *cdc2^+^* and *erg32^+^*, relative to the DMSO-treated cells and 3-MB-PP1-treated *cdk9^as^* cells. This indicates redundant roles of Cdk9 and H2Bub1 in maintaining nucleosome occupancy at these genes. The effect on nucleosome occupancy was less pronounced at two subtelomeric genes and at an intergenic region near *cen3*, suggesting that it was driven by RNAPII transcription (Supplementary Figure S8A). Histone H3 acetylation (at K9 and K14), when corrected for changes in H3 occupancy, was also not significantly affected by either Cdk9 inhibition or *htb1-K119R* alone but was synergistically increased by the combination of the two. Increased relative histone acetylation also coincided with increased antisense expression at *cdc2^+^* and *erg32^+^*in *hrp3Δ, set2Δ* and *cph1Δ* cells (Supplementary Figures S8B and C). These data suggest that Cdk9 and H2Bub1 collaborate to recruit Clr6-CII, promote histone deposition and deacetylation, and suppress antisense transcription genome-wide.

### Loss of H2Bub1 alters transitions in the RNAPII transcription cycle

**Figure 7.**
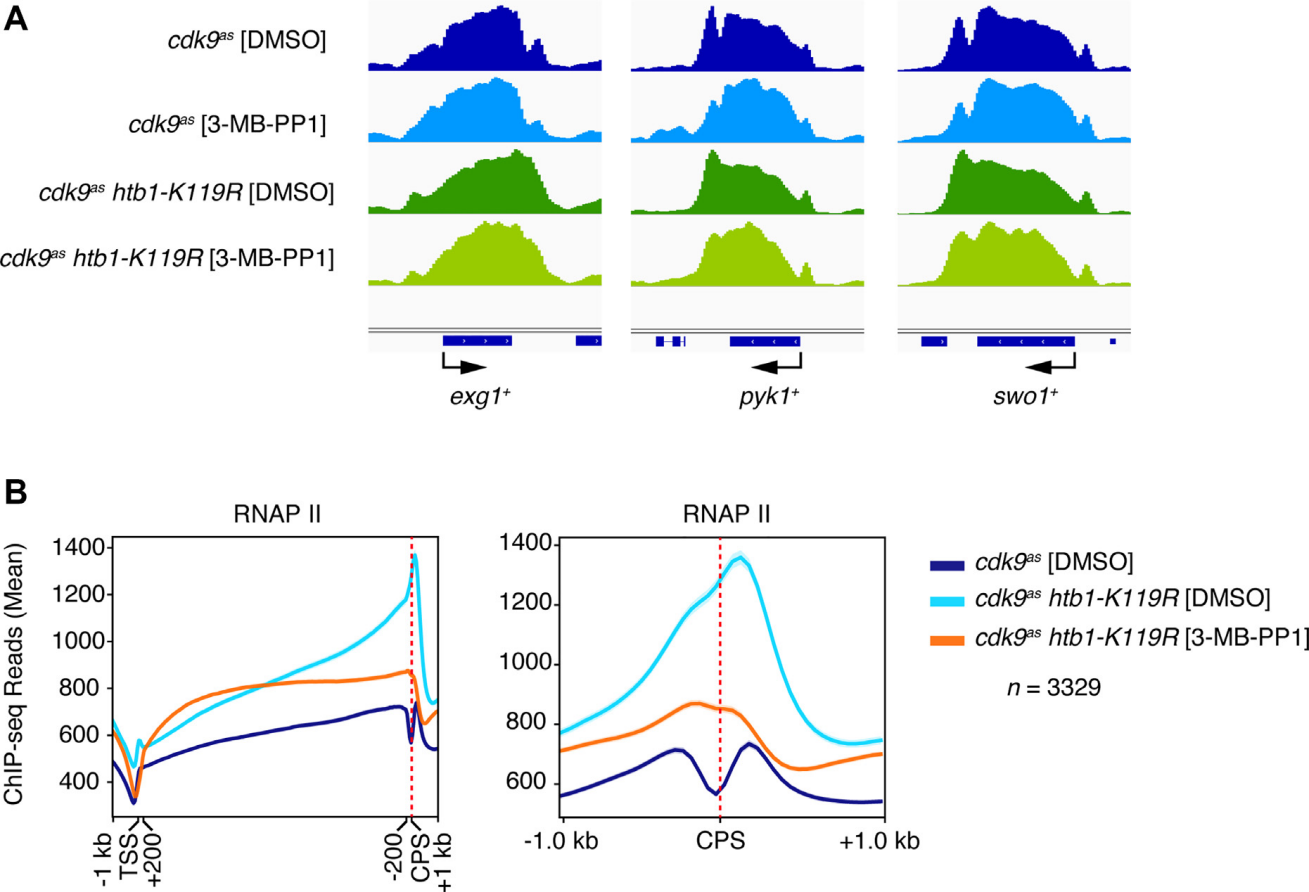
Loss of H2Bub1 alters RNAPII distribution around the CPS. (**A**) Browser tracks show RNAPII distribution on representative genes in the indicated strains treated with 3-MB-PP1 or DMSO. (**B**) Metagene plots show RNAPII distribution across the entire gene, with scaling of regions between 200 bp downstream of TSS to 200 bp upstream of CPS (left); and around the CPS (±1 kb, unscaled; right), for 3329 genes.

The 3′ shift of RNAPII upon H2Bub1 loss, which was evident genome-wide by both ChIP-chip (23) and ChIP-seq analyses (Figure 5A), prompted us to look more closely at *htb1-K119R* effects on RNAPII distribution in individual gene tracks (Figure 7A). A consistent feature of ChIP-seq profiles in control cells—DMSO-treated *cdk9^as^* or wild-type—is a bimodal distribution of RNAPII near the 3′ ends of genes, with peaks or prominences on either side of a steep trough centered over the CPS (26,44). This pattern was also apparent in Spt5 ChIP-seq profiles, in both fission yeast (44) and budding yeast (52), but has not been explained. A possible reason for such a discontinuity in ChIP data is epitope occlusion, i.e., a conformational change, possibly associated with transcript cleavage, which impeded antibody interactions with target proteins. Similarly positioned troughs appear in PRO-seq profiles in both budding and fission yeast, however, making this explanation unlikely (18,33,44). Strikingly, the trough was largely or completely effaced in H2Bub1-deficient cells, both in the absence or presence of Cdk9 activity (*cdk9^as^ htb1-K119R* + 3-MB-PP1 or DMSO, respectively), as is evident in single-gene tracks (Figure 7A) and a CPS-centered metagene analysis (Figure 7B). (This would seem to rule out another trivial explanation, i.e. ‘drop-outs’ of sequencing reads around the CPS due to nucleotide bias.) In a diagnosis of exclusion, we surmise that the trough might indicate transient acceleration of RNAPII as it approaches, and deceleration once it passes, the CPS. Our results suggest, furthermore, that these changes in elongation rate are attenuated on chromatin lacking H2Bub1.

## DISCUSSION

Perturbations of chromatin structure due to passage of the transcription complex carry an inherent risk of inappropriate access by initiation factors. Accordingly, enzymes and factors that regulate elongation also act to suppress cryptic initiation, in part by influencing chromatin structure in gene coding regions (27). Here we have shown that Cdk9 and H2Bub1 each promote the function of the Clr6-CII complex in *S. pombe*. Combined Cdk9 inhibition and H2Bub1 loss lead to a genome-wide mismatch between Clr6-CII and RNAPII, associated with decreased nucleosome occupancy, impaired histone deacetylation and the de-repression of antisense transcription.

ChIP-seq analysis showed that H2Bub1 and pSpt5 co-occupy gene coding regions genome-wide, consistent with a shared function during RNAPII elongation (23). We also observed that the distributions of these two modifications differed around the TSS and CPS regions: H2Bub1 levels (normalized for total histone) rose 5′ of pSpt5 levels near the TSS and fell 3′ of pSpt5 levels near the CPS. The uncoupling of H2Bub1 and pSpt5 proximal to the TSS may reflect a Cdk9-independent mechanism for H2Bub1 formation at the initiation stage of transcription. In mammalian cells, RNF20/40 can be recruited to target genes through interaction with the Mediator complex (53). This could also account, at least in part, for the dependence of pSpt5 on H2Bub1 (23). At the CPS, reduction in the pSpt5/Spt5 ratio occurs 5′ of a similar reduction in H2Bub1/H2B ratio; the latter effect is a result of nucleosome removal. Loss of H2Bub1 through histone turnover near the CPS was observed in another recent study (54). This suggests that Spt5 dephosphorylation might trigger chromatin remodeling events leading to H2Bub1 removal at the 3′ ends of genes.

Although Cdk9 is not strictly required for RNAPII release from the promoter-proximal pause in fission yeast (18), our data suggest that it is needed to suppress antisense transcription preferentially at paused genes, on which elongation might be more stringently regulated. Pause sites in *S. pombe* coincide with the dyad axis of the +1 nucleosome, typically within genes that harbor a defined intragenic nucleosomal array (33). H2Bub1 and Clr6-CII have been implicated in the establishment of nucleosome organization in coding regions (11,29,55), which might in turn dictate location and duration of pausing. Furthermore, Clr6-CII recruitment was particularly affected in 5′ regions of pause-regulated genes that depend on Cdk9 to suppress antisense transcription.

In contrast to Cdk9 inhibition, the *htb1-K119R* mutation caused accumulation of antisense transcripts preferentially at genes with low basal levels of H2Bub1. The basis for this dichotomy is still undetermined, but a possible scenario is suggested by the observation that Cdk9 inhibition leads to an ∼3-fold slowing of RNAPII elongation (18), which we suspect would afford greater opportunity for cryptic initiation. This effect might be more pronounced at highly transcribed genes, which also typically have high H2Bub1 (23). The *htb1-K119R* mutant, in contrast, is likely to have a less pronounced effect on RNAPII elongation rates, suggesting that increased antisense transcription might arise in this setting by another mechanism, such as defective nucleosome positioning or stability at genes with limiting H2Bub1 levels.

Induced degradation of Spt5 has been shown to promote antisense transcription in *S. pombe* (26). Here, we implicate the major Spt5 kinase Cdk9, and chromatin-modifying enzymes that act downstream of pSpt5, in suppression of antisense transcription. Several of our results argue against a linear Cdk9-pSpt5-H2Bub1 pathway restraining antisense transcription, however, and hint at greater complexity. For example, the super-induction of antisense transcription by Cdk9 inhibition, in H2Bub1-deficient cells with an *spt5(T1A)_7_* allele, suggests that Cdk9-mediated suppression of cryptic initiation occurs through at least one pathway that does not depend on Spt5 phosphorylation at Thr1 within its CTD repeats. The lack of an effect due to *spt5(T1A)_7_* alone, moreover, implies that those mechanisms can compensate for loss of pSpt5 in H2Bub1-proficient cells.

Studies of the budding yeast Rpd3S complex identified multiple determinants of its localization and function, including histone modifications and components of the transcription machinery (32,56–59). H2Bub1 had not previously been linked to the function of an Rpd3S-related HDAC *in vivo*, although the human ortholog of one Clr6-CII subunit, MRG15, interacts with H2Bub1 *in vitro* (60). Here we uncover a cooperative effect of Cdk9 and H2Bub1 on recruitment and function of fission yeast Clr6-CII in gene bodies, suggesting that H2Bub1 is an important chromatin feature recognized by Rpd3S/Clr6-CII-type HDACs.

H2Bub1 has been functionally linked to CHD-type ATP-dependent chromatin remodeling factors (61,62). In *S. pombe* these factors repress antisense transcription by enforcing nucleosome positioning, a function also ascribed to H2Bub1 (11,50). At the loci we tested, antisense transcription increased upon deletion of a remodeler in this class, *hrp3^+^*. The effects were additive with those of H2Bub1 loss or Cdk9 inhibition, however, suggesting separate pathways, whereas Clr6-CII acts downstream of H2Bub1 and Cdk9.

While this work was under review, Murawska et al. reported a role for H2Bub1 in suppressing antisense transcription in *S. pombe*, consistent with the findings reported here (54). Their study indicated that loss of H2Bub1 increased antisense transcription by allowing an aberrant activity of the FACT histone chaperone complex. This was suggested to stem from a direct effect of H2Bub1 on FACT, but our data suggest that it could reflect impaired function of Clr6-CII. Evidence of opposing functions of FACT and Rpd3S in *S. cerevisiae* is consistent with this possibility (63).

Despite the fact that Cdk9 activity and H2Bub1 affect Clr6-CII occupancy and antisense suppression on their own, it is only when both are compromised that evidence of reduced Clr6-CII function—increased relative histone acetylation and reduced histone occupancy—is detected genome-wide. This may be because Cdk9 inhibition or *htb1-K119R* alone impairs an HDAC-independent, nucleosome-stabilization function of Clr6-CII, which is only required for antisense suppression at a subset of genes (55). Inactivation of both Cdk9 and H2Bub1 pathways might reduce Clr6-CII functions in gene coding regions below the threshold needed to suppress antisense transcription.

Reduced Cdk9 activity impaired recruitment of Set2 in ChIP-qPCR experiments whereas loss of H2Bub1 did not, consistent with previous results (23,31,40). Cdk9 inhibition had gene-specific effects on antisense derepression caused by *set2Δ*, suggesting that it acts through both Set2-dependent and Set2-independent pathways. The epistasis relationship between *htb1-K119R* and *set2Δ* implies that H2Bub1 and H3K36me3 function in the same pathway. This pathway may well lead to Clr6-CII; this would predict that H2Bub1 would enhance nucleosome binding activity of Clr6-CII, as has been described for H3K36me3.

Cdk9 inhibition increased RNAPII occupancy in the ∼500-bp region downstream of the TSS; *htb1-K119R* caused re-localization of RNAPII towards the 3′ ends of genes, with a discrete peak over the TSS. These changes confirm and extend our previous RNAPII ChIP-chip results, and are consistent with PRO-seq data indicating an ∼500-bp window downstream of the TSS in which Cdk9 activity is critical (18,23). The localized accumulation of RNAPII near the TSS and CPS in *htb1-K119R* cells suggests that H2Bub1 modulates RNAPII rate and/or processivity at specific positions within genes. H2Bub1 seems to be needed for an acceleration by RNAPII upstream of the CPS. Downstream of the CPS, RNAPII decelerates, concomitant with Spt5 dephosphorylation and exchange of H2Bub1 for unmodified H2B. Since the *htb1-K119R* strain does not have obvious termination defects, the function of this shift in RNAPII dynamics is unclear. Interestingly, recent micrococcal nuclease (MNase)-seq analyses in *htb1-K119R* cells demonstrated that nucleosome stability was generally compromised within gene coding regions (in agreement with previous data in *S. cerevisiae*; (11)), but was slightly enhanced compared to wild-type immediately downstream of the TSS and upstream of the CPS (54). This suggests that the effects of H2Bub1 on chromatin structure and RNAPII distribution are functionally related. Determining how these effects relate to H2Bub1-dependent regulation of the Clr6-CII complex or antisense transcription will require further investigation.

Cdk9 inhibition produced qualitatively similar changes in RNAPII distribution in *cdk9^as^* and *cdk9^as^ htb1-K119R* cells, i.e. a shift toward the 5′ end. An important question for future studies is whether the seemingly opposite effects of H2Bub1 loss and Cdk9 inhibition on RNAPII distribution reflect partial versus complete loss of Cdk9 function, respectively, or genuinely opposing functions of Cdk9 activity and H2Bub1 in elongation. Although the impact of Cdk9 inhibition on RNAPII distribution within genes was similar in *htb1^+^* and *htb1-K119R* backgrounds, the global increase in RNAPII occupancy in coding regions was only observed in *htb1^+^* cells. We attribute this difference to a global decrease in nucleosome occupancy in 3-MB-PP1-treated *cdk9^as^ htb1-K119R* cells, which we surmise counteracts the effects of reduced Cdk9 activity on RNAPII elongation rate.

Under no condition was altered RNAPII distribution accompanied by a concomitant re-distribution of Clr6-CII. This argues that the pattern of Clr6-CII association with transcribed chromatin is not strictly dictated by the underlying RNAPII pattern, although we cannot rule out that interactions between Clr6-CII and RNAPII that occur in wild-type cells are blocked by Cdk9 inhibition or *htb1-K119R*. Biochemical studies of Rpd3S indicate that the complex has a robust nucleosome binding activity even in the absence of histone modifications (56); this could be its primary determinant for stable binding to transcribed chromatin. Association with the RNAPII elongation complex promoted by Cdk9 or H2Bub1 may serve to recruit the complex transiently to sites of transcription, which sets the stage for more stable association to occur.

The mammalian ortholog of Rpd3S/Clr6-CII is the Sin3B complex, which localizes to transcribed genes and is a potential therapeutic target in pancreatic cancer (30,64). A question emerging from our studies is whether Sin3B, like Clr6-CII, is responsive to Cdk9 or H2Bub1. HDAC inhibitors induce histone hyperacetylation in gene coding regions and intergenic regions in mammalian cells, suggesting that HDACs such as Sin3B may function in a manner analogous to Clr6-CII (65). Study of the mechanisms that regulate HDAC function in gene coding regions may thus be relevant to further clinical development of HDAC inhibitors.

## DATA AVAILABILITY

The processed sequencing files from RNA-seq are available on NCBI BioProject ID:382240. ChIP-seq files have been submitted to the NCBI Gene Expression Omnibus (GEO; http://www.ncbi.nlm.nih.gov/geo/) (Accession numbers: GSE115682, GSE102590, GSE31071).

## Supplementary Material

gkaa474_Supplemental_Files
