# Supplementary material for: Cdk9 and H2Bub1 signal to Clr6-CII/Rpd3S to suppress aberrant antisense transcription
Source: Nucleic Acids Res. 2020 Jun 4;48(13):7154–68. doi: 10.1093/nar/gkaa474 (PMC7367204; doi:10.1093/nar/gkaa474)
Supplement: gkaa474_Supplemental_Files [file gkaa474_supplemental_files.zip › Supplementary Figure Legends.docx]

**Supplementary Figure Legends**

**Figure S1**. Cdk9 inhibition and *htb1-K119R* synergistically increase antisense transcripts throughout the genome. Plot of the percentage of protein-coding genes in the indicated categories, defined by a minimum 2-fold change from the wild-type expression level in each condition as determined by analysis with DESeq (see Materials and Methods).

**Figure S2**. Validation of synergistic effects of Cdk9 inhibition and *htb1-K119R* on select antisense transcripts. **(A)** Strains (JTB362, JTB299, JTB508, JTB86) were treated with DMSO (-) or 3-MB-PP1 (+) and the indicated antisense transcripts, as well as the control *tfa2^+^* transcript, were quantified by conventional RT-PCR. **(B)** Northern blot analysis of the *cdc2^+^* antisense transcript on total RNA derived from the indicated strains treated with DMSO (-) or 3-MB-PP1 (+) for 1 hour.

**Figure S3.** H2Bub1 and pSpt5 occupancies for genes at which antisense levels are induced by Cdk9 inhibition, *htb1-K119R*, or both. **(A)** Scaled metagene representations of pSpt5/Spt5 or H2Bub1/H2B ratios from transcription start site (TSS) to cleavage and polyadenylation site (CPS) for genes with increased antisense transcripts. Genes were clustered into three groups based on total pSpt5/Spt5 occupancy. **(B)** Top: Metagene representations of H2Bub1/H2B ratios for the indicated groups of genes, plotted from the indicated distances (in kilobases) relative to the TSS. Bottom: Heat map representations of the same gene groups sorted by gene length (increasing from top to bottom). **(C)** As in (B) for pSpt5/Spt5 ratios.

**Figure S4**. Antisense induction by Cdk9 inhibition and *htb1-K119R* is not sensitive to neighbouring genes. **(A)** Metagene profiles of expression levels derived from the RNA-seq data were compared for all genes. **(B)** As in (A) for groups of genes with no neighbour, genes 5’ of a divergently oriented gene, or genes 5’ of a tandemly oriented gene.

**Figure S5**. Epistasis analysis of antisense regulation at additional candidate genes and sense transcript control. **(A)** Strand-specific RT-qPCR quantified sense transcript levels at *cdc2^+^* locus in the indicated strains. Value for the wild-type strain was set to 1 (n=3). **(B)** and **(C)** Antisense transcript levels at *hrp1^+^* and *hem2^+^* were quantified by RT-qPCR in the indicated strains, respectively. Value for the wild-type strain was set to 1 (n=3). **(D)** and **(E)** Antisense transcript levels at *hrp1^+^* and *hem2^+^* genes in the indicated *cdk9^as^* strains, respectively, after treating cells either with 3-MB-PP1 or mock-treated with DMSO. Value for the DMSO-treated *cdk9as* strain was set to 1 (n=3).

**Figure S6**. Metagene plots of Clr6-CII occupancy on genes that require Cdk9 or H2Bub1 for antisense suppression. **(A)** Immunoblot analysis of Pst2-myc, H2Bub1 and total histone H3 in the indicated strains. **(B)** Left to right: Metagene plots of RNAPII, Pst2, and their log_2_-ratio (Pst2/RNAPII) upstream (-0.5 kb) and downstream (+2 kb) of the TSS, on genes with increased antisense transcription after Cdk9 inhibition (*n* = 522), in the indicated strains after treatment with 3-MB-PP1 or DMSO. **(C)** and **(D)** As in (B) for groups of genes with increased antisense transcription due to loss of H2Bub1 (*n* = 537), or loss of both H2Bub1 and Cdk9 activity (*n* = 2767), respectively.

**Figure S7.** Cdk9 activity, but not H2Bub1, is required for Set2 recruitment to chromatin and H3K36me3. **(A)** ChIP-qPCR was performed with the indicated antibodies (right) and analyzed with primer pairs at the indicated positions in *cdc2*^+^. “-“ and “+” denote treatment with DMSO or 3-MB-PP1, respectively. FLAG IPs from strains expressing Set2-FLAG (top panels) were normalized to IPs from untagged strains. Error bars denote S.D. from 3-4 independent experiments. “*” denotes a significant (*p*<0.05; unpaired t-test) difference between DMSO and 3-MB-PP1. **(B)** As in (A) for *erg32^+^.*

**Figure S8**. Set2 and Clr6-CII decrease histone acetylation at the *cdc2^+^* and *erg32^+^* loci. **(A)** ChIP-qPCR shows nucleosome occupancy at subtelomeric genes and in an intergenic region in the indicated strains after treatment with DMSO or 3-MB-PP1. (**B and C**) ChIP of acetylated histone H3 was performed in the indicated strains (JTB362, JTB142, JTB414, and JTB636), quantified by qPCR with primer pairs in the indicated genes, and normalized to total H3 occupancy. The H3Ac/H3 ratio for the wild-type strain was set to 1. Error bars denote S.D. from 2-3 independent experiments.

S
