# Supplementary material for: Cdk9 and H2Bub1 signal to Clr6-CII/Rpd3S to suppress aberrant antisense transcription
Source: Nucleic Acids Res. 2020 Jun 4;48(13):7154–68. doi: 10.1093/nar/gkaa474 (PMC7367204; doi:10.1093/nar/gkaa474)
Supplement: gkaa474_Supplemental_Files [file gkaa474_supplemental_files.zip › supptable1NAR2020.docx]

**Table S1**. *S. pombe* strains used in this study.

| **Strain (JTB#)** | **Genotype** | **Source** |
| --- | --- | --- |
| 80-2 | *set1∆::kanMX6 ade6-M216 h+* | Ref. 10 |
| 97 | *htb1-K119R::kanMX6 ade6 leu1-32 h-* | Ref. 10 |
| 142 | *h- set2∆::hphMX6 ade6* | Ref. 66 |
| 299 | *cdk9as::kanMX6 leu1-32 ura4-D18 his3-D1 ade6-M210 h+* | Ref. 24 |
| 331 | *brl2∆::hphMX4 ade6-M210 h-* | Ref. 10 |
| 362 | *leu1-32 ura4-D18 his3-D1 ade6-M210 h+* | Ref. 24 |
| 386 | *brl2∆::hphMX4 ade6 cdk9as::kanMX6 h? leu1? ura4?* | Ref. 24 |
| 408 | *cdk9as::kanMX6 set1∆::kanMX6 h?* | This study |
| 414 | *h+ cph1∆::kanMX4 ade6-M216 ura4 D18 leu1-32* | This study |
| 425 | *cdk9as::natMX6 leu1-32 ura4-D18 his3-D1 ade6-M210 h+* | This study |
| 443 | *spt5(7)::ura4+ cdk9as::natMX6 ade6 ura4-D18 leu1-32 h-* | This study |
| 444 | *spt5-T1A(7)::ura4+ cdk9as::natMX6 ade6 ura4-D18 leu1-32 h-* | This study |
| 508 | *cdk9as::natMX6 htb1-K119R::kanMX6 ade6 leu1? ura4? h?* | Ref. 24 |
| 618 | *h? hrp3∆::LEU2 htb1-K119R::kanMX6 ade6 leu1-32 ura4?* | This study |
| 636 | *h- hrp3∆::LEU2 ade6 leu1-32 ura4?* | K. Ekwall |
| 154 | *h? set2∆::hphMX6 htb1-K119R::kanMX6 ade6* | This study |
| 640 | *cph1∆::hphMX6 htb1-K119R::kanMX6 ade6 leu1-32 ura4-D18 h?* | This study |
| 736 | *spt5-T1A(7)::ura4+; cdk9as::natMX6 htb1-K119R::kanMX6 ade6 leu1-32 ura4-D18 his3? h?* | This study |
| 738-2 | *spt5(7)::ura4+; cdk9as::natMX6 htb1-K119R::kanMX6 ade6 leu1-32 ura4-D18 his3? h?* | This study |
| 621 | *h+ alp13∆::hphMX6 ade6-M216 ura4 D18 leu1-32* | This study |
| 622 | *h+ cph1∆::hphMX6 ade6-M216 ura4 D18 leu1-32* | This study |
| 752 | *htb1-K119R::hphMX6 ade6 leu1-32 ura4D-18 h+* | This study |
| 442 | *cdk9as::natMX6 set2∆::hphMX6 leu1? ura4-D18 his3? ade6 h?* | This study |
| 505 | *cdk9as::natMX6 cph1∆::kanMX4 ade6 ura4-D18 leu1-32 his3? h?* | This study |
| 639 | *cdk9as::kanMX6 hrp3∆::LEU2 ade6 leu1-32 ura4? his3? h?* | This study |
| 837 | *h- pst2-myc::natMX6* | E. Hidalgo PG76 |
| 838 | *cdk9as::kanMX6 pst2-myc::natMX6* *h?* | This study |
| 842 | *cdk9as::kanMX6 pst2-myc::natMX6* *htb1-K119R::hphMX6 h?* | This study |
| MS265 | *spt5::13myc::kanMX6 htb1-FLAG::natMX6 leu1-32 ura4-D18 his3-D1 ade6-M21X h+* | Ref. 24 |
| 873 | *rrp6**∆::kanMX6 h+* | This study |
| 989 | *h- leu1-32 ura4-D18 dcr1∆::hygMX6* | 1. Morillon |
| 990 | *h- exo2∆::kanMX4* | 1. Morillon |
