# Supplementary material for: Cdk9 and H2Bub1 signal to Clr6-CII/Rpd3S to suppress aberrant antisense transcription
Source: Nucleic Acids Res. 2020 Jun 4;48(13):7154–68. doi: 10.1093/nar/gkaa474 (PMC7367204; doi:10.1093/nar/gkaa474)
Supplement: gkaa474_Supplemental_Files [file gkaa474_supplemental_files.zip › supptable2NAR2020.docx]

| **Primer** | **Sequence (5’-3’)** | **Application** |
| --- | --- | --- |
| cdc2-1 | ATCATTCTCGCATCTCTATTA | ChIP qPCR (with cdc2-2) |
| cdc2-2 | ATTCTCCATTGCAAACCACTA |  |
| cdc2-3 | AAAAATTGGGGAAGGTAG | antisense-specific primer, qRT-PCR (with cdc2-4) |
| cdc2-4 | ATAAACAACGCCATAGGT |  |
| cdc2-5 | CCAGCTAGTGAACGGTGTA | ChIP qPCR (with cdc2-6) |
| cdc2-6 | AATTGCCTTCTTTGTCTATCA |  |
| cdc2-9 | GAAGTATGGCCTGGAGTCACG | ChIP qPCR (with cdc2-10) |
| cdc2-10 | ATGGTAATGCAAAATCTT |  |
| erg32-1 | CGCCGGAGAACAATCGAGA | ChIP qPCR (with erg32-2) |
| erg32-2 | AGCGTACTGCAAAACGACATC |  |
| erg32-3 | GGCCAGAAGGCACCCTAAAT | ChIP qPCR (with erg32-4) |
| erg32-4 | AGCAAGAAACCAAGGAGCTGT |  |
| erg32-5 | CGCTGTCGTAAATGGTGCTG | ChIP qPCR (with erg32-6), antisense-specific primer, qRT-PCR (with erg32-6) |
| erg32-6 | GCGCTGCTCTTGCAATTTCT |  |
| act1-1 | GGTTGCTCAATGTTATCCGTTTC | ChIP qPCR (with act1-2), |
| act1-2 | TGATAAAGCCACACACAGCGTTA |  |
| act1-5 | CCACTATGTATCCCGGTATTGC | ChIP qPCR (with act1-6), qRT-PCR |
| act1-6 | CAATCTTGACCTTCATGGAGTT | sense-specific primer |
| aes1-1 | ACAGATGTGCTCAACCTCGT | ChIP qPCR (with aes1-2) |
| aes1-2 | CTTCGGCTTTCGAACGAGTC |  |
| grt1-3 | GCAGTGGGAAGGTTATTGGA | ChIP qPCR (with grt1-3) |
| grt1-4 | ACCGTGTTCTCCGTAACCAG |  |
| osIRC3F | GGATCATTATTAAAGTACTAGATG | ChIP qPCR (with osIRC3R) |
| osIRC3R | CGAGAGTAAAAATGATCTGTAATG |  |
| hem2FW | GGAAGAGTTTTTGGGTCCTCTC | ChIP qPCR (with hem2RV) |
| hem2RV | GTGACGAATTTCTTAACGGC |  |
| hrp1FW | GAGGATCATCCTTCTAGAAC | ChIP qPCR (with hrp1RV) |
| hrp1RV | GCTTAGTGATGGACTTAGTGG |  |

**Table S2**. Oligonucleotide primers used in this study.
