# Supplementary figures and images for: Cdk9 and H2Bub1 signal to Clr6-CII/Rpd3S to suppress aberrant antisense transcription
Source: Nucleic Acids Res. 2020 Jun 4;48(13):7154–68. doi: 10.1093/nar/gkaa474 (PMC7367204; doi:10.1093/nar/gkaa474)

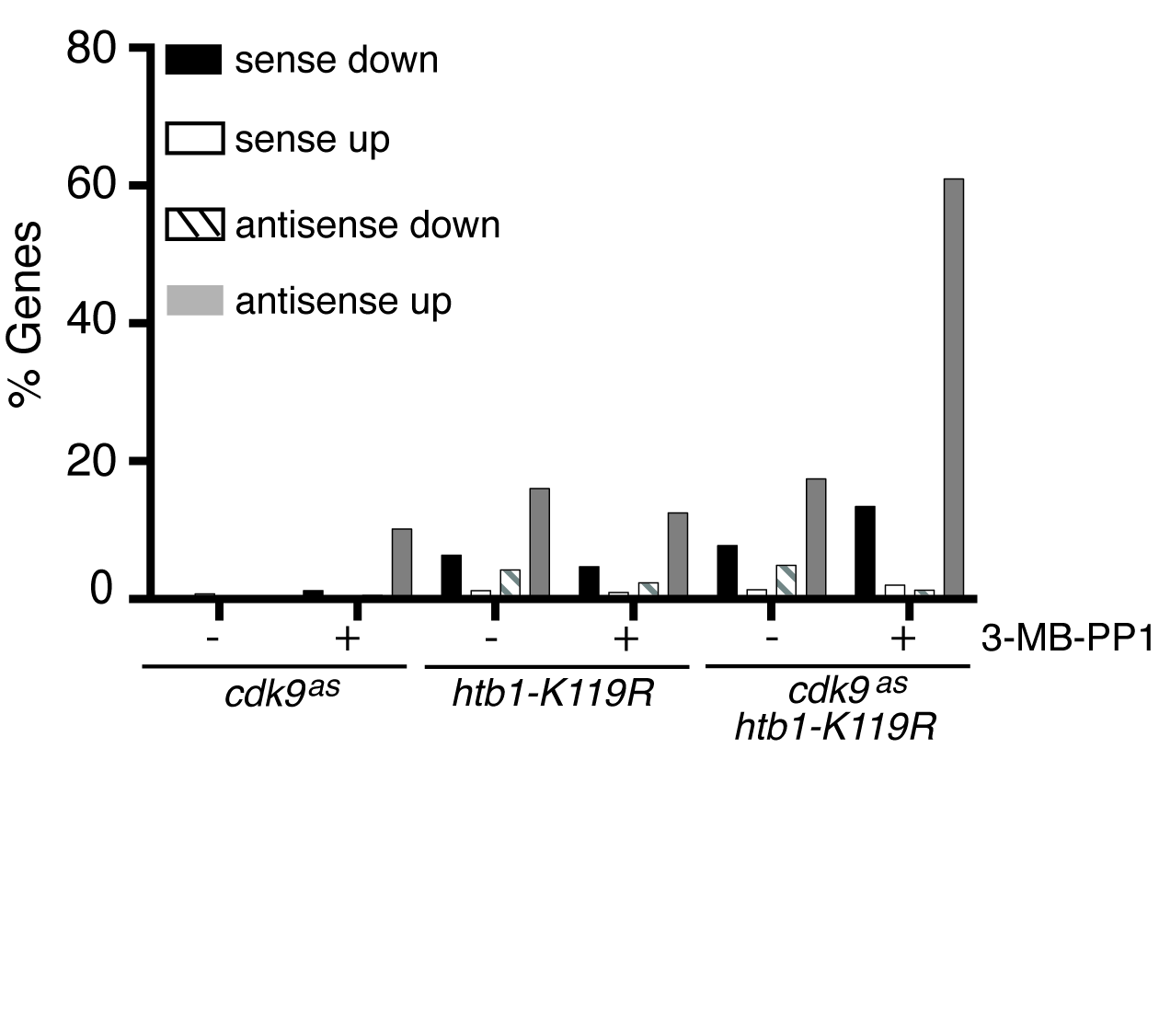

Supplement: gkaa474_Supplemental_Files [file gkaa474_supplemental_files.zip › Fig S1.tif]

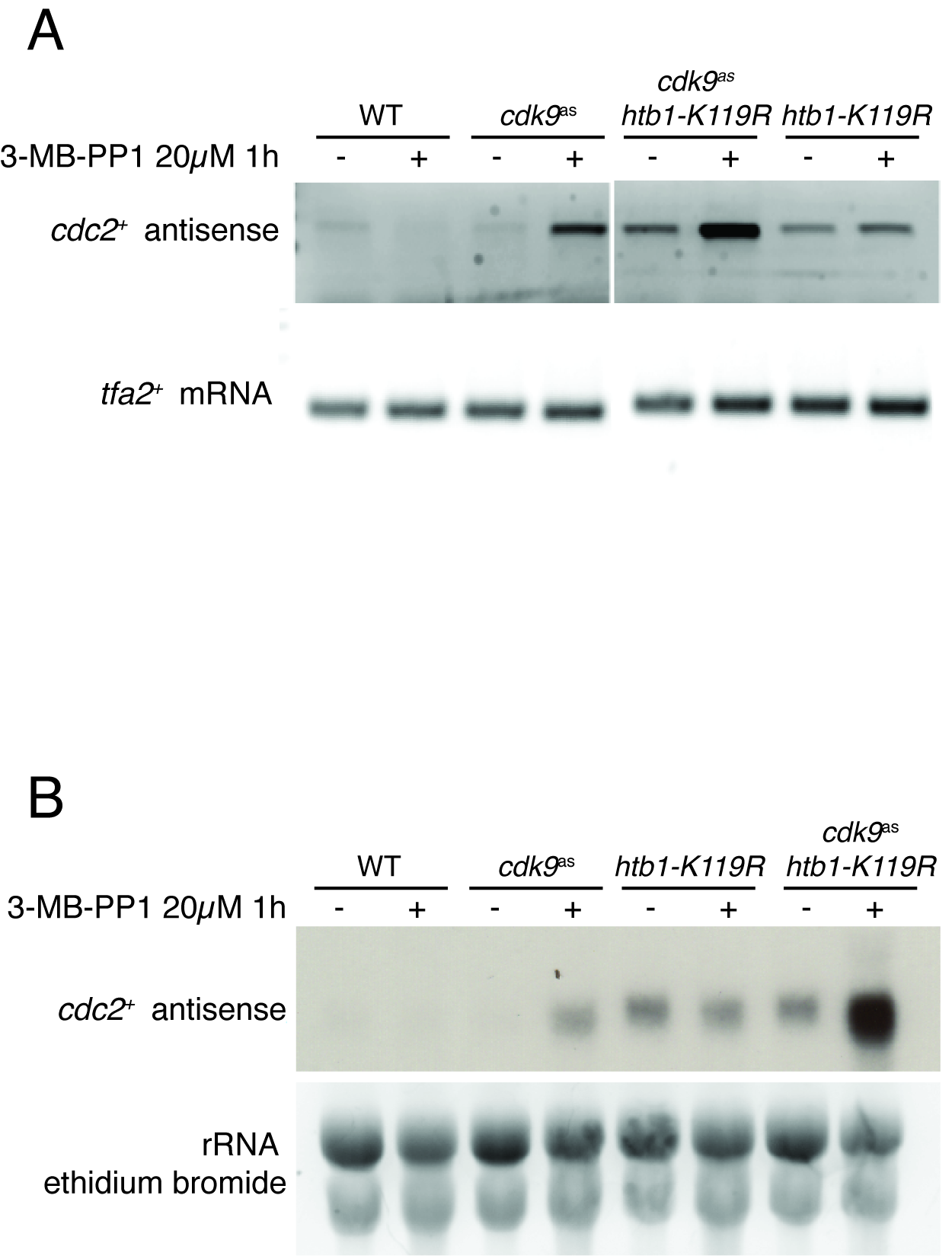

Supplement: gkaa474_Supplemental_Files [file gkaa474_supplemental_files.zip › Fig S2.tif]

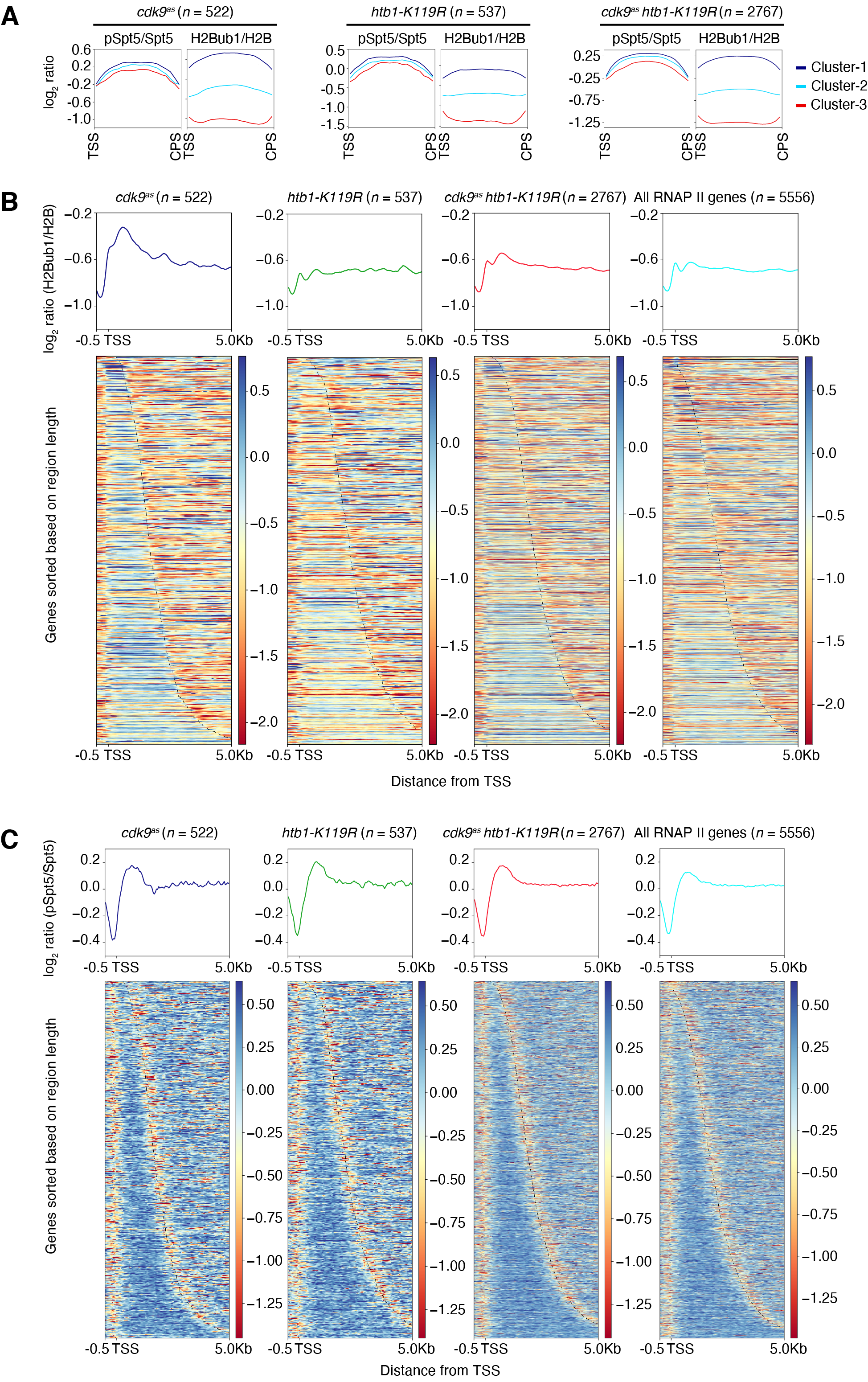

Supplement: gkaa474_Supplemental_Files [file gkaa474_supplemental_files.zip › Fig S3.png]

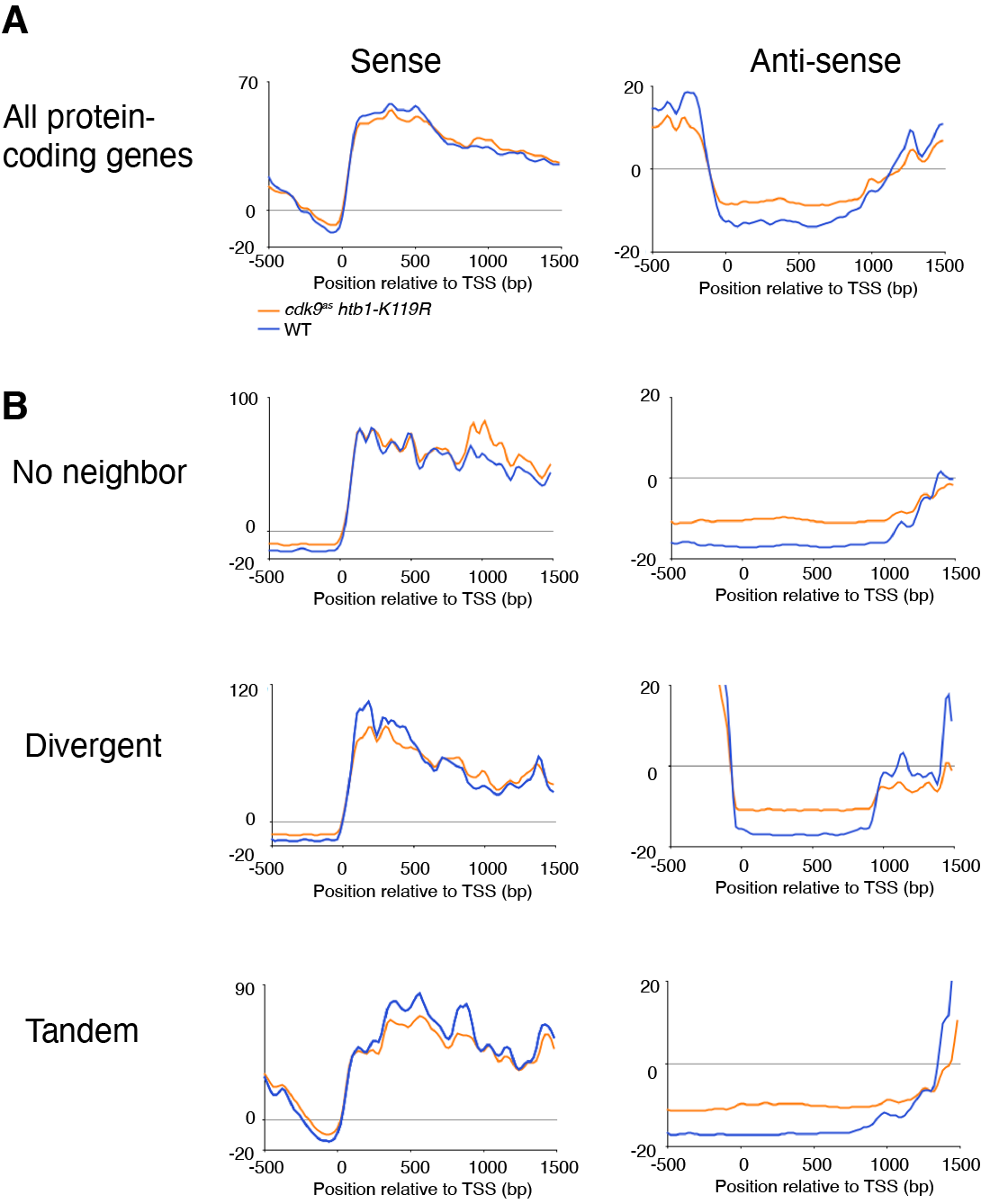

Supplement: gkaa474_Supplemental_Files [file gkaa474_supplemental_files.zip › Fig S4.png]

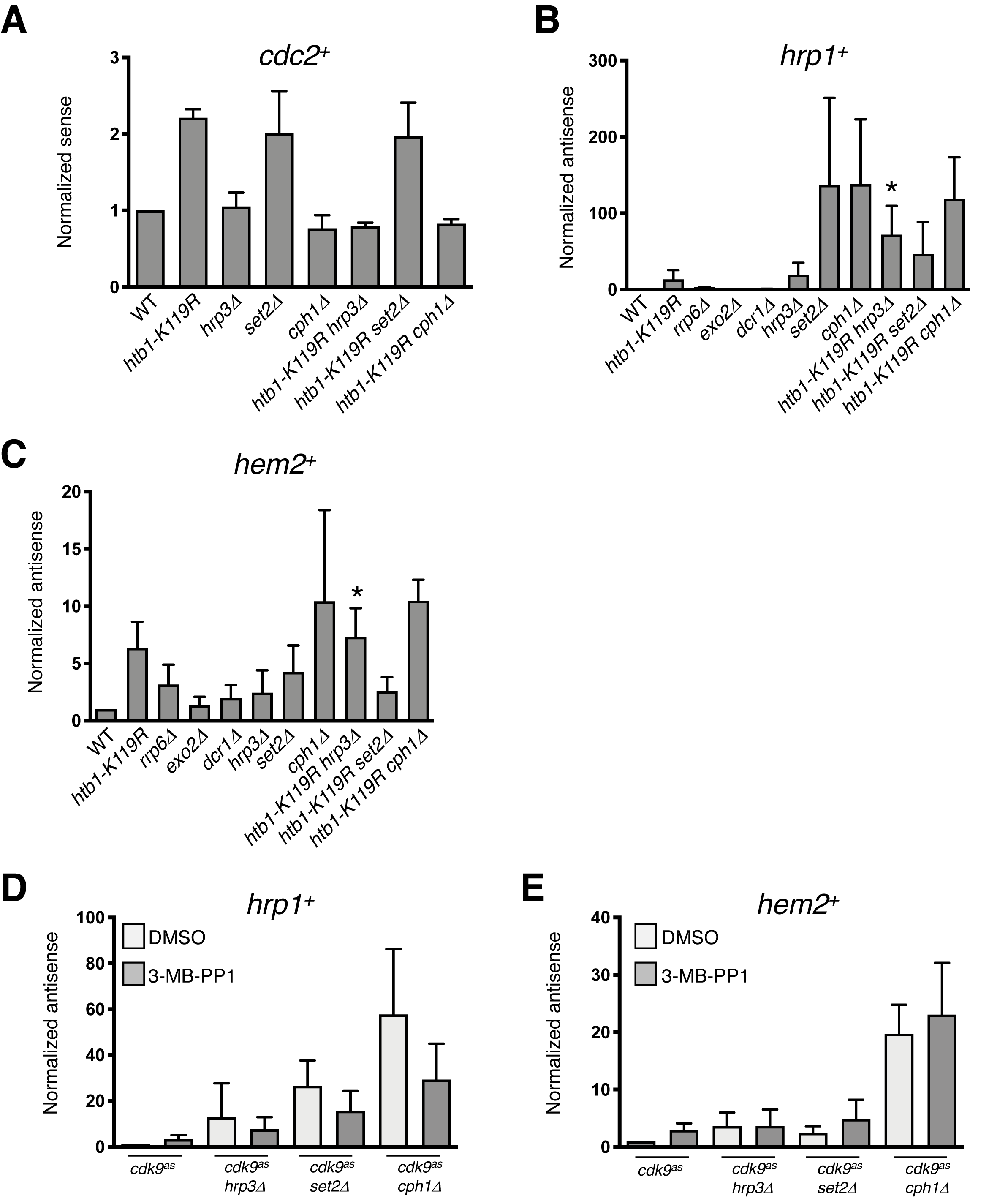

Supplement: gkaa474_Supplemental_Files [file gkaa474_supplemental_files.zip › Fig S5.tif]

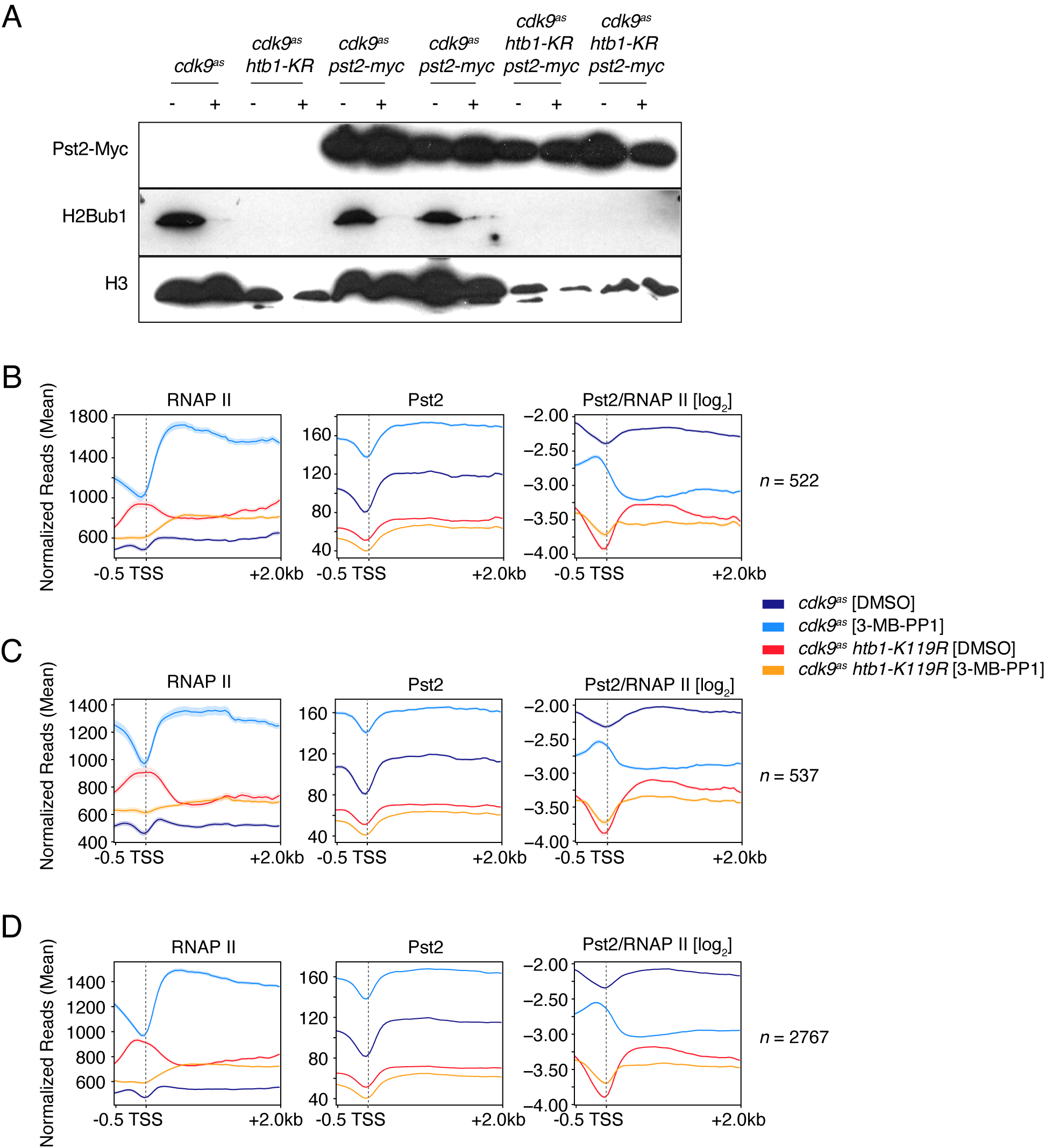

Supplement: gkaa474_Supplemental_Files [file gkaa474_supplemental_files.zip › Fig S6.tif]

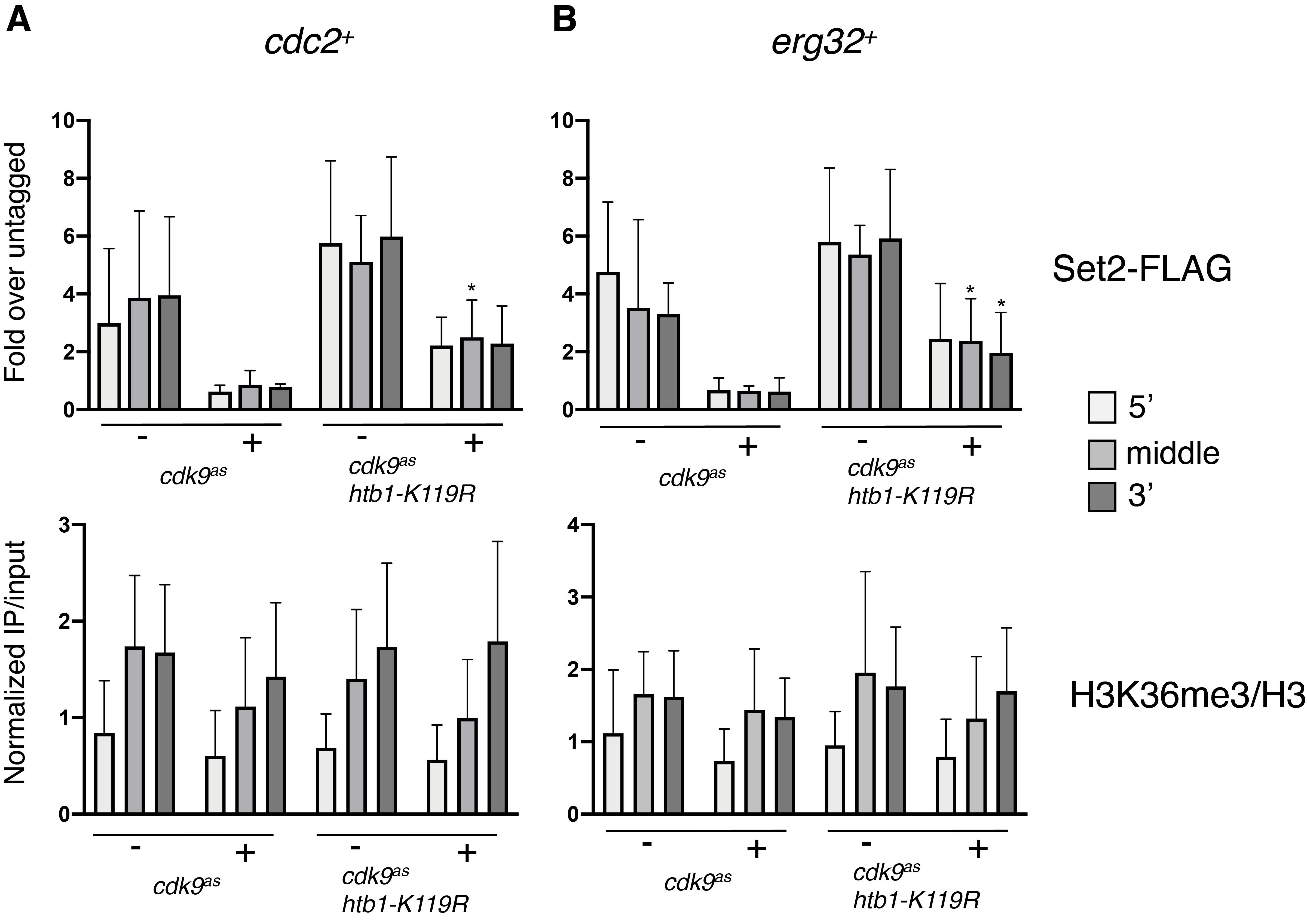

Supplement: gkaa474_Supplemental_Files [file gkaa474_supplemental_files.zip › Fig S7.png]

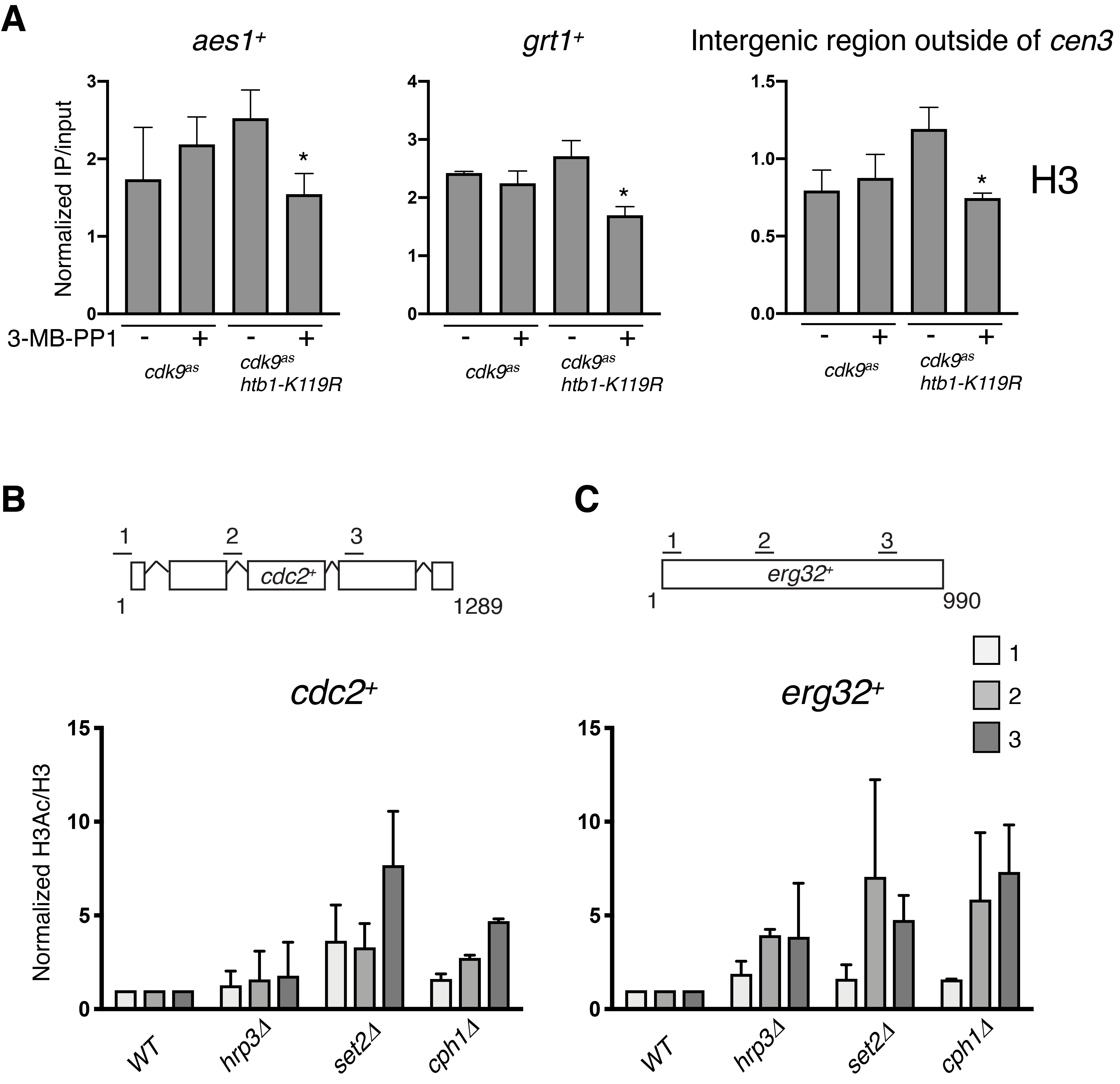

Supplement: gkaa474_Supplemental_Files [file gkaa474_supplemental_files.zip › Fig S8.png]
